# Comparing the Nitration of Nanostructured CNF and Other Cellulose Forms for Energetic Applications

**DOI:** 10.1021/acsomega.5c12095

**Published:** 2026-01-26

**Authors:** Ana Carolina Marotti Dias, Cesar Liberato Petzhold, Leena Pitkänen, Maurício Ferrapontoff Lemos, Fernando Cunha Peixoto

**Affiliations:** † Seção de Engenharia Química, Instituto Militar de Engenharia, Praça General Tibúrcio 80, 22290-270 Rio de Janeiro, Brazil; ‡ Instituto de Química, 28124 Universidade Federal do Rio Grande do Sul, Av. Bento Gonçalves 9500, 91501-970 Porto Alegre, Brazil; § Department of Bioproducts and Biosystems, 174277 Aalto University, P.O. Box 16300, 00076 Aalto, Finland; ∥ Instituto de Pesquisas da Marinha, Grupo de Materiais, Rua Ipiru, s/n, 21931-090 Rio de Janeiro, Brazil

## Abstract

This work focuses on the nitration of nanocellulose to obtain products with potential advantages over traditional ones obtained from linter cellulose (LC). The material used was a sample of cellulose nanofibrils (CNF) commercially available as an aqueous suspension with a 3% solid content. The main objective of the research was to develop the reaction conditions for nitration, including the preparatory methods for the cellulose raw material, so that the properties on the nanometric scale were maintained. The dehydration of the CNF to concentrate the fibrils in suspension was investigated using different methods: gravity and vacuum filtration, centrifugation, oven, and freeze-drying. The nitration experiments were carried out with dehydrated CNF following a protocol based on methodologies reported in the literature. For comparative purposes, commercial samples of cotton LC and microcrystalline cellulose (MCC) were also evaluated. A complete characterization of the structural and thermal properties of the cellulose materials and their nitration products was carried out using the following techniques: size-exclusion chromatography, scanning electron microscopy, infrared spectroscopy, X-ray diffraction, simultaneous thermogravimetric analysis, and elemental analysis. The results of the analysis revealed that the chemical structure of the cellulose chains is neither altered by the removal of water nor by the temperature and pressure conditions. However, the agglomeration of the fibrils is favored due to the interconversion of hydrogen bonds between cellulose–water and cellulose–cellulose, as in the heating and freezing treatments, making the redispersion of the nanofibers impossible. Only dehydration by vacuum filtration was suitable for preparing CNF for the reaction to obtain nitrocellulose. Although the resulting degree of substitution was not yet suitable for energetic applications, it was possible to obtain nitrated products from the commercial cellulosic materials under study. Comparing the structural and thermal properties of the nitrated CNF with those of LC and MCC nitration products obtained under identical conditions, the results corroborated the work in the literature and the theories developed so far. Therefore, the study demonstrated the potential viability of the synthesis methodology developed for the nitration of cellulose of different origins, dimensions, and morphological types.

## Graphical abstract

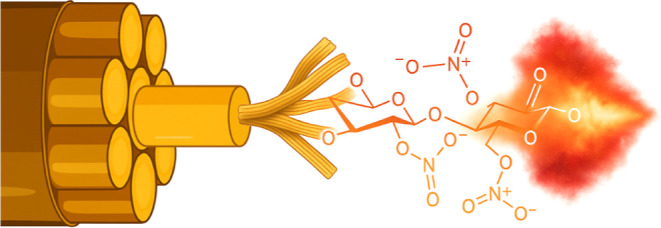

## Introduction

Cellulose is one of the most abundant organic substances in nature, constituting about 40% of the Earth’s carbon reserves. It is found in plants, some marine animals (tunicates), invertebrates, algae, protozoa, fungi, and even bacteria.^1^ In plants, it is the main structural component of the cell wall and is extracted via chemical and/or mechanical processes.^2,3^

Plant cellulose is tightly associated with hemicelluloses and lignin, forming lignocellulosic biomass.^4^ Hemicelluloses are short, branched polysaccharides, while lignin is an amorphous aromatic polymer. Cellulose is isolated through delignification, followed by alkaline treatments, yielding alpha-cellulose, a highly reactive fraction.

Cellulose consists of β-1,4-linked anhydro-d-glucose units (AGU) with the molecular formula C_6_H_10_O_5_, connected in long, linear chains with degrees of polymerization (DP) ranging from 300 to 15,000, depending on the source.^2,3,5^ Higher DPs correlate with a higher molar mass and strength.

As an abundant raw material, it serves as a renewable source for obtaining specialty chemicals through structural modifications and/or functionalization.^2–4^ However, the intrinsic variability of its origin and processing methods directly affects the length and structure of cellulose chains, leading to considerable fluctuations in the properties and chemical behavior between different batches.^5^

Ultimately, the lack of uniformity in cellulose feedstock significantly impacts the physical, chemical, mechanical, and ballistic properties of nitrocellulose (NC)-based propellants for guns, missiles, and rockets.^6^ In manufacturing plants, blending steps of different NC batches are performed to mitigate these effects. However, this attempt to homogenize final product characteristics only produces apparent results, intermediate to the mixed lots, since the process is limited to the macroscopic level.^7^

In energetic materials, particle morphology significantly affects the thermal decomposition phenomenon, directly influencing explosion and combustion behavior.^8^ The modification of NC morphology through physicochemical methods—such as supercritical antisolvent, electrospinning in organic solvent, and recrystallization—demonstrates performance benefits. In these processes, the elemental composition of NC remains unchanged, retaining the nitrogen content and molecular structure of the starting cellulose.^9–14^

Reported results^11–14^ indicate increased burn rates and reduced sensitivity to friction and impact. Nanoscale NC exhibits higher reactivity than micron-sized NC. Additionally, the onset of thermal decomposition is advanced, as shown by lower onset temperatures in thermal analysis curves.^9,14^ This occurs because reaction mechanisms are governed by chemical kinetics at the nanoscale, rather than mass transport limitations.^9,15^ The increased surface area lowers activation energy, accelerating overall decomposition and energy release.^6,13^

Despite these advantages, the downsizing methods involve extreme conditions, such as high temperatures, pressures, and/or electric fields, which increase costs and risks, especially considering NC’s flammability and explosiveness. Moreover, the final nitrogen content of NC—which, aside from kinetic correlations, is independent of size reduction operations—remains a predominant factor in applications involving energetic materials.

As alternative energetic polymer precursors, micro- and nanostructured celluloses—so-called nanocelluloses—have emerged as promising raw materials.^16^ Recent reviews have emphasized nanocellulose as a strategic material for military and defense-related applications, highlighting its potential use in energetic systems, protective materials, and advanced functional composites due to its high strength-to-weight ratio and tunable surface chemistry.^17^ In nanotechnology, the production and use of cellulose nanocrystals (CNCs) and nanofibrils (CNF) have attracted research interest. CNCs are the crystalline segments of cellulose microfibrils obtained via selective degradation of amorphous regions by enzymatic or chemical means. CNF are obtained through mechanical disintegration of cellulose fibers.^2,3,18^ In this context, bacterial nanocellulose has also been explored as a nitration feedstock, yielding nitrate derivatives with distinct molecular weight distributions, crystallinity, and thermal behavior when compared to plant-derived celluloses.^19^

The nitration products of these materials have been investigated for improving the environmental profile, cost, safety, and performance of NC for explosive formulations. Generally, improved structural organization and thermal stability have been observed.^6,20,21^ Additionally, the increased surface area provides more reactive sites and alters thermodynamic and kinetic parameters.^18,22^

The heterogeneity and spontaneous degradation of conventional NC pose significant challenges for safety and ballistic performance. Although stabilizing agents can mitigate autocatalytic decomposition, they do not prevent the breakdown of the nitrate bonds. Moreover, over time, mechanical degradation of NC fibers leads to microfractures, increasing the burning surface area and risking overpressure or explosion in confined systems.^23,24^

Beyond conventional formulations, nitrated bacterial cellulose has also been incorporated into energetic nanocomposites, demonstrating its applicability as both a propellant and explosive matrix for military-oriented systems.^25^

These stability issues demand innovative approaches, and nanostructuring appears as a viable route. However, traditional nanosizing methods are operationally intensive and do not allow tailoring of the nitrogen content. Therefore, new sources—such as micro- and nanocelluloses—may offer a safer, more controllable pathway to functional NC derivatives.

Nanocelluloses, such as CNCs and CNF, are derived from cellulose through mechanical or chemical processing. They exhibit high surface area, stiffness, crystallinity, and modifiable surface chemistry, enabling applications in composites, coatings, and as precursors for functional materials.^1,2^

From a sustainability perspective, recent assessments have discussed the environmental impact of nanocellulose-based nitrated polymers, indicating potential advantages over conventional nitrocellulose in terms of resource efficiency, life-cycle considerations, and greener energetic material design.^26^

Their large surface area allows for diverse chemical modifications, enabling tailored reactivity and performance. This is particularly valuable for producing nitrated derivatives with a controlled nitrogen content and improved stability.

NC is synthesized by nitrating cellulose with nitric and sulfuric acid mixtures. It serves as a matrix and fuel in smokeless powders and solid rocket propellants.^27,28^ Its dual structural and energetic functions are critical in energetic formulations.

Manufacturing NC requires precise control of acid ratios, reaction time, temperature, and cellulose source. This ensures the desired nitrogen content and physical characteristics while maintaining safety in handling.

Nitration occurs through electrophilic substitution, where hydroxyl groups react with the nitronium ion (NO_2_^+^). Accessibility of these groups is affected by crystallinity, surface area, and the presence of lignin or hemicelluloses.

The nitrogen content classifies NC by application: lower % N (<10.7%) for films; medium (11.1–12.2%) for explosives; high (>12.5%) for propellants.^20^ Higher % N increases the energy but reduces the stability.

NC solubility in organic solvents depends on the degree of substitution. Its gelatinization behavior governs how it is processed into sheets, granules, or other forms for energetic applications.

Nanostructured celluloses provide more accessible sites for nitration, enhancing efficiency and control over nitrogen content. Nitrated CNF and CNCs show improved thermal stability, reactivity, and uniformity, representing promising alternatives for high-performance NC materials.^6,20–22^

More recently, surface-selective nitration strategies under mildly acidic conditions have been reported for cellulose nanofibers, enabling preferential functionalization of accessible hydroxyl groups while preserving the crystalline core of the nanofibers.^29^

Motivated by the growing interest in tailoring energetic materials through structural control rather than solely compositional modification, the present work explores whether nanostructured cellulose can act as a viable precursor for morphology-driven nitrocellulose design. Beyond fundamental aspects of reactivity and accessibility, this approach is guided by the perspective that nitrocelluloses derived from nanocelluloses may enable new processing routes, including molded grains and additive manufacturing strategies (intended for special rocket motor applications requiring complex geometries), where rheology, homogeneity, and geometric freedom are critical. In this context, understanding how precursor morphology and dehydration history affect nitration efficiency constitutes a necessary step toward assessing the feasibility of nanocellulose-based nitrocelluloses for advanced propellant architectures.

Additionally, the aging of nitrocellulose-based propellants involves, in addition to the release of nitrous groups, modifications of their polymeric backbone. Therefore, the use of nanocelluloses as nitration feedstocks would yield products that could be regarded as representing the limiting case of this second aging mechanism, ensuring the reproducibility of their ballistic behavior throughout their service life.

Therefore, the present work aimed to analyze the nitration of commercially available nanostructured celluloses by characterizing different feedstocks and the respective products obtained, using gel permeation chromatography, scanning electron microscopy (SEM), infrared spectroscopy, X-ray diffraction (XRD), and elemental analysis.

## Methodology

This section presents the materials and equipment used as well as the laboratory methods adopted to fulfill the specific objectives of this work.

An overview of the methodology is illustrated in the flowchart in Figure 1, which summarizes the set of activities carried out to fulfill each stage.

**Figure 1:**
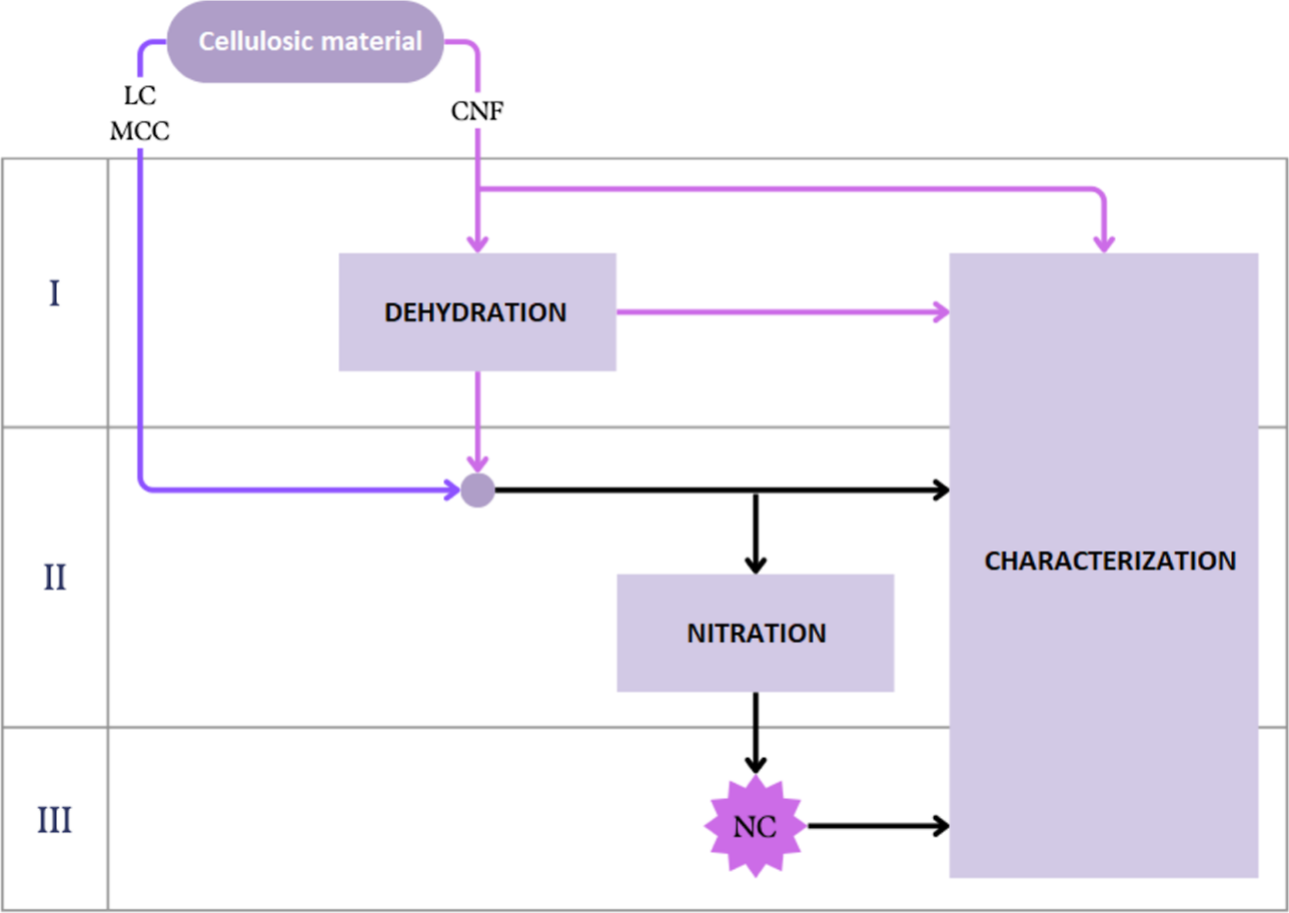
Flowchart of the work development stages.

Throughout all stages, characterization activities were carried out—starting materials (I and II) and nitrated products (III)—whose techniques and operational parameters will be detailed. The dehydration of CNF in stage I includes water removal methods, which were investigated to concentrate the nanofibers in a suspension. The dehydration of CNF is particularly challenging due to its high water retention capacity, arising from the extensive hydrogen-bonding network between fibrils and the large specific surface area of the nanostructure. This strong affinity for water leads to gel-like behavior, making the removal of water without causing irreversible fibril aggregation or loss of nanostructural characteristics a critical step. In stage II, the nitration activity includes the experiments conducted with dehydrated CNF; the procedures and reaction conditions for obtaining products will be described. For comparative purposes, the following commercial cotton celluloses were selected: linter (LC), the main raw material for nitration in the explosives industry, and MCC, widely used in literature studies. Both were characterized and nitrated identically to CNF. The evaluation of the nitrated products was carried out through the analyses performed in stage III.

### Cellulosic Material

The main raw material studied was CNF “Valida L, 3%” (3% cellulose fibrils suspended in water) supplied by Sappi Limited (Maastricht, The Netherlands), which consists of cellulose nanofibrils, produced by processing wood fibers through mechanical treatments. When dispersed in water, they form an extensive three-dimensional network via hydrogen bonding and mechanical entanglement. They are commercialized with 3 or 8% solid content in aqueous suspension and are used to stabilize formulations of cosmetics, paints and varnishes, and concrete additives, among other applications. Purified and bleached LC was donated by the Presidente Vargas Factory of the Brazilian Military Material Industry (FPV/IMBEL), and MCC 310697 in powder form (20 μm; batch MKCV0964) was purchased from Sigma-Aldrich. The purified linter batch intended for NC manufacture was subjected to mechanical and chemical treatments, namely, bleaching with sodium hypochlorite solution, acidification in an autoclave with sulfuric acid solution, defibrillation, centrifugation, and drying.

### Characterization Techniques

#### Size-Exclusion Chromatography

The SEC technique was performed at the time of donation of the CNF sample used in this study to evaluate the MMD of the celluloses in stage I. CNF and LC samples were collected directly from the original packages and sent to sealed containers without prior treatment. MCC was acquired later and not analyzed.

The instrumentation—shown consists of the following modules: SEC Dionex UltiMate 3000 by Thermo Scientific, coupled with multiangle light scattering (MALS) detectors Viscotek/Malvern, model SEC/MALS 20, and refractive index (DRI) detector Shodex RI-101. Four PLgel 20 μm MIXED-A columns by Agilent, size 300 × 7.5 mm, were used with dimethylacetamide (DMAc) containing 0.9% lithium chloride (LiCl) as the mobile phase at 0.75 mL/min.

The samples were dissolved in DMAc/LiCl (9%), diluted in a 1:10 ratio, and filtered through a 0.22 μm syringe filter prior to analysis. Solvent exchange with water, acetone, and finally DMAc was performed. The CNF sample was lyophilized prior to dissolution. The value of ∂n/∂c of 0.136 mL/g was used for both celluloses in DMAc/LiCl (0.9%). The injection volume was 100 μL. Column and detector temperatures were 22 and 25 °C, respectively. MALS and DRI detector constants were determined using polystyrene standards with narrow (*M*_w_ = 96,000 g/mol, *D̵* = 1.04) and broad (*M*_w_ = 248,000 g/mol, *D̵* = 1.73) molecular mass distributions. The procedures adopted for sample preparation, analysis, constant determination, and detector calibration were based on the methodology described in the literature.^5,30^

#### Scanning Electron Microscopy

Surface morphology was analyzed by SEM. CNF analysis in Stage I was conducted with a TESCAN MIRA 4 LMU SEM (LowVac Mode UniVac), equipped with a Schottky Field Emission Gun (FEG), secondary electron (SE) Everhart–Thornley detector, and retractable backscattered electron (R-BSE) detector with a YAG (yttrium aluminum garnet) crystal. Prior to scanning, the CNF sample was oven-dried and sputter-coated with gold to avoid charging effects. Image processing was performed by using TESCAN Essence software.

In Stages II and III, SEM analysis was conducted using a QUANTA FEG 250, with BRUKER XFlash 6-60 and e-Flash detectors. Samples were deposited on carbon double-sided tape and coated with approximately 10 nm of gold using a Leica EM ACE 600 high-vacuum sputter coater for 30 min. Analyses were performed at a 5 kV acceleration voltage on both systems.

#### Infrared Spectroscopy

Chemical functionality was analyzed by Fourier-transform infrared spectroscopy using the attenuated total reflectance mode (FTIR–ATR) on a PerkinElmer Spectrum 100 spectrometer. Analyses were carried out using a universal ATR accessory, in the wavenumber range of 650–4000 cm^–1^, with 16 scans and a resolution of 4 cm^–1^ per sample. Thermo Scientific Spectrum 10.5.1 software was used to control the instrument and process the data. Background spectra were collected before each measurement and automatically subtracted from the sample spectra.

#### X-ray Diffraction

XRD analyses were performed using a Malvern PANalytical X’Pert PRO Multi-Purpose diffractometer with cobalt radiation (Co Kα_1_ = 1.7903 Å). Operating parameters were a 40 kV accelerating voltage, a 40 mA tube current, a 2θ scan from 5 to 80°, a step size of 0.0197°, and a 97.920 s count time per step.

Stainless-steel sample holders like the one in Figure 2 were used, with the cavity lined with a plastic film to load the samples. Data processing was carried out using X’Pert High Score software, including smoothing, peak identification, background adjustment, and theoretical profile fitting, with calculations of parameters of interest from the resulting diffractograms, such as peak area and intensity.

**Figure 2:**
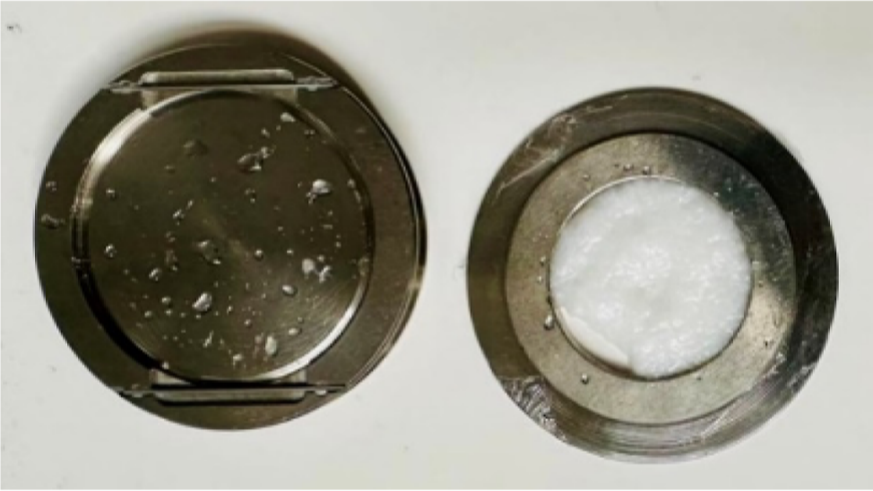
Stainless-steel sample holder.

The crystallinity index (CrI) was calculated using the methods of Segal^31^ and Ruland-Vonk,^32,33^ according to eqs 1 and 2.

The first method is based on peak intensities corresponding to the (200) plane, which accounts for the crystalline portion, and the minimum between peaks related to planes (110) and (200), corresponding to the amorphous portion.^34^ The second is based on the ratio between the total area of amorphous contribution and the full scan area above the background of the diffractogram.^35^

1 $$CrI_{Segal}=\frac{\left(I_{200}-I_{am}\right)}{I_{200}}$$

2 $$CrI_{RV}=1-\frac{\Sigma \left(S_{am}\right)}{\Sigma \left(S_{cr}\right)+\Sigma \left(S_{am}\right)}$$

where *I*_200_ and *I*_am_ are the intensities of the (200) peak and amorphous scattering, respectively; *S*_cr_ and *S*_am_ are areas related to crystalline and amorphous regions, respectively.

### Thermal Analyses

The degradation profile and thermal stability were investigated by simultaneous thermogravimetric analysis (STA), using a Shimadzu DTG-60H instrument. The technique combines thermogravimetric (TGA) and differential thermal (DTA) analyses in a single instrument for simultaneous measurement. TGA measures changes in sample mass, while DTA measures the temperature difference between a sample and a reference material over a temperature range. In addition to the decomposition profile, information about the exothermic or endothermic nature of the events can be obtained.

The analyses were conducted under a nitrogen atmosphere with a flow rate of 50 mL/min, over a temperature range from room temperature to 600 °C, with a heating rate of 10 °C/min. A lidless platinum pan was used to load the samples. The Shimadzu LabSolutions TA:Postrun 1.00 software was used for data processing and calculation of parameters of interest from the resulting thermograms such as mass loss and characteristic temperatures associated with thermal events.

### Elemental Analysis

The elemental analysis technique was employed in stage III to determine the percentage composition of elements present in the nitrated products obtained in stage II, in order to assess the nitrogen content. The analyses were carried out using a PerkinElmer 2400 Series II CHNS/O organic elemental analyzer. Samples of the respective starting cellulosic materials were also analyzed.

### Dehydration Methods

The dehydration of CNF to concentrate the fibrils in suspension was investigated through the different methods outlined in Figure 3. Initially, 3.0 g samples were collected directly from the original packaging, placed in Falcon-type conical tubes, and homogenized in a Benchmark Scientific vortex mixer, model BV1000, in continuous mode for 5 min.

**Figure 3:**
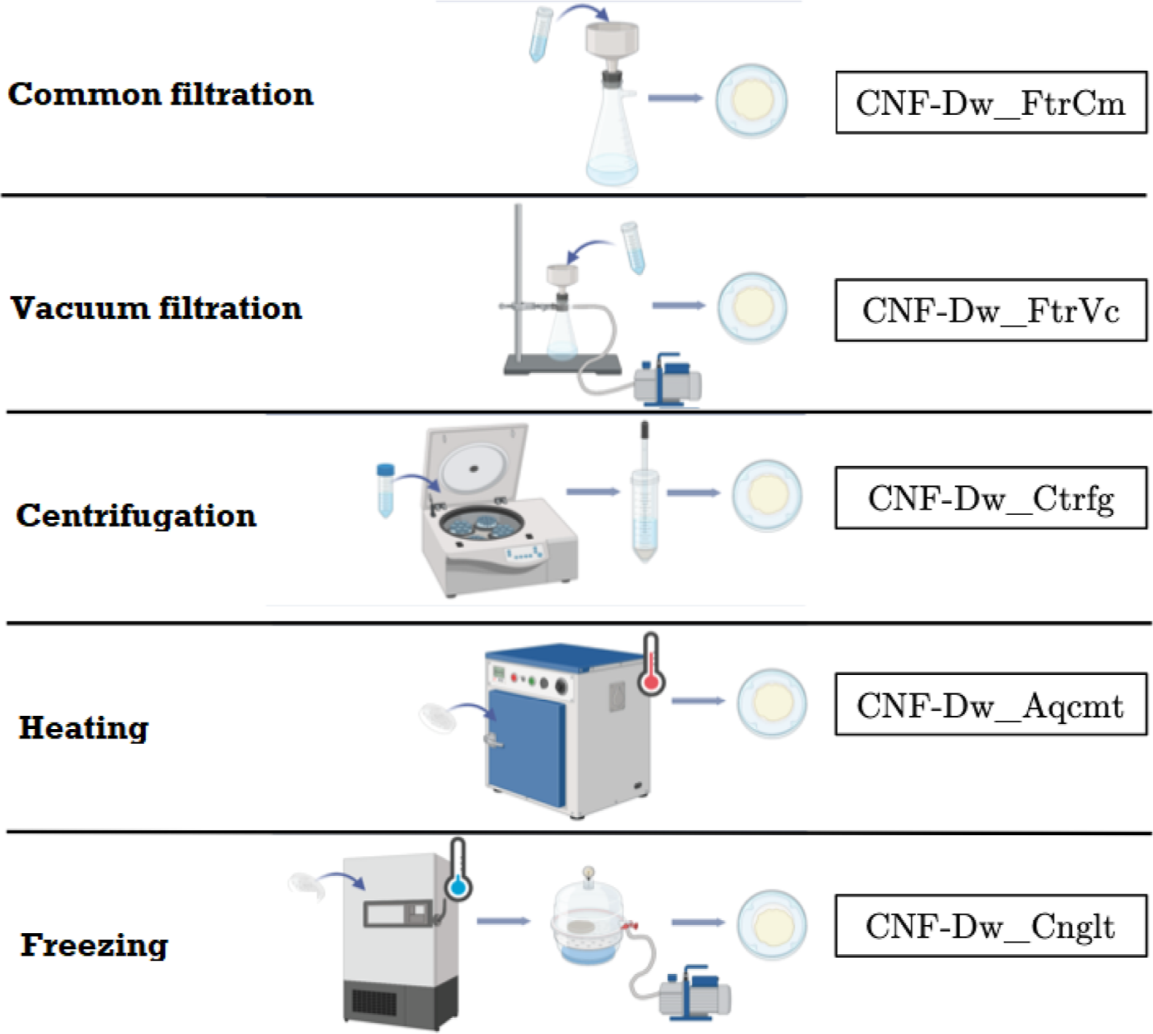
Dehydration methods for CNF samples.

Filtrations—both gravity and vacuum—were conducted using a porcelain Büchner funnel with ashless filter paper (grade 42, Whatman) coupled to a vacuum flask. A Jet Pump WEG motor pump (W56X210) was used to apply the vacuum. Centrifugation was performed using a NOVA Instrument benchtop centrifuge at 3000 rpm for 5 min, followed by removal of the aqueous phase (top) with a Pasteur pipet.

Samples for dehydration by heating and freezing were placed in Petri dishes to avoid thick layer formation. The first method was performed in a sterilization and drying oven at 110 °C for 3 h. In the second method, the sample-containing plate was placed in a freezer at −20 °C and subsequently in a desiccator connected to an Edwards RV8 high-vacuum pump for 24 h.

The dehydrated samples were analyzed by FTIR–ATR, XRD, and STA according to the methodology already described. The codes used for identification refer to the respective treatment received, as presented in Figure 3.

### Cellulose Nitration

The cellulosic raw materials were nitrated using a sulfonitric solution—whose composition was defined based on methodologies and results reported in the literature—and reaction conditions adapted from refs 16, 21, and 36 . The nitrated products obtained were analyzed by SEM, FTIR–ATR, XRD, and STA under the same conditions as the raw materials, according to the methodology described in previous sections.

The nitrogen content (% N) represents the mass percentage of nitrogen in the NC, being a key factor for its application, with an impact on chemical and physical properties such as viscosity, solubility, stability, and energetic characteristics.^37^ The degree of substitution (*z*) is directly related to % N, as well as the carbon and hydrogen content, % C and % H, using the representative chemical formula $C_{12}H_{14}O_{4}{\left(OH\right)}_{6-z}{\left(ONO_{2}\right)}_{z}$ ,^28^ which lead to eqs 3–5

3 $$\%\;C=\frac{144}{324+45z}100\%$$

4 $$\%\;N=\frac{14z}{324+45z}100\%$$

5 $$\%\;H=\frac{\left(20-z\right)}{324+45z}100\%$$

Once experimental data on % N, % C, and % H are available, one can conduct a reconciliation procedure as described in eq 6

6 $$\min_{z}{\left(\frac{144}{324+45z}-\frac{\%\;C}{100\%}\right)}^{2}+{\left(\frac{14z}{324+45z}-\frac{\%\;N}{100\%}\right)}^{2}+{\left(\frac{\left(20-z\right)}{324+45z}-\frac{\%\;H}{100\%}\right)}^{2}$$

to find (*z*) given by eq 7

7 $$z=-\frac{4\left(7290\cdot \%\;C+1377\cdot \%\;H-5103\cdot \%\;N-332,500\right)}{5\left(810\cdot \%\;C+153\cdot \%\;H-567\cdot \%\;N+17,980\right)}$$

If cellulose were fully nitrated, that is, *z* = 6, the nitrogen content would be 14.14%, but industrially, values up to 13.5% are achievable. The higher the % N value, the more unstable the NC becomes. Above the 11–12% range, NC is classified as an explosive, exhibiting highly flammable characteristics. Below 10%, NC is classified as collodion and is used in the paint industry.^38^

The reagents used for nitration were sulfuric acid 98% (P.A., 1.40 kg/L, Vetec), nitric acid 65% (P.A., 1.84 kg/L, Vetec), and distilled water prepared in the laboratory. In the stabilization stage, anhydrous sodium carbonate (P.A., Proqumios) was used. The protocol followed consisted of the steps schematically presented in Figure 4 and in the following sections. All procedures were carried out inside a fume hood.

**Figure 4:**
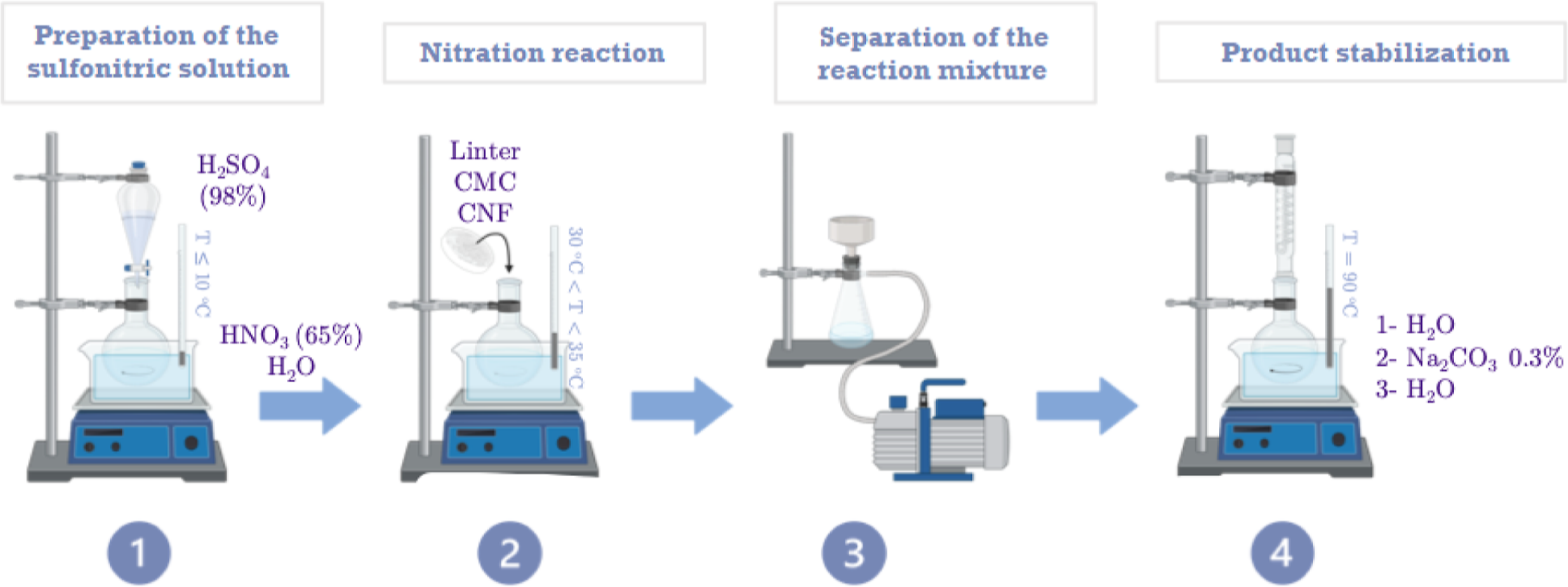
Schematic representation of the nitration protocol steps.

In the experiments carried out, the quantities of reagents were calculated based on the composition presented in Table 1, with a 1:2 molar ratio of nitric acid to sulfuric acid. The total volume of the reaction mixture was 25 mL/g cellulose.

**Table 1:** Composition of the Sulfonitric Solution

component	individual molar concentration (mol/L)	volumetric addition ratio (v/v)	mass fraction in solution (%)
H_2_SO_4_	18.4	0.54	61.1
HNO_3_	14.4	0.37	20.9
H_2_O	-	0.09	18.0

### Preparation of the Sulfonitric Solution

In a two-necked round-bottomed glass flask equipped with a magnetic stirrer, distilled water was first added. The system was mounted on an IKA C-MAG HS 7 stirring hot plate with an ice-water bath at a temperature of 6 ± 1 °C, monitored using a thermometer. Then, nitric acid—measured with a graduated cylinder—was added to the flask under constant magnetic stirring at 500 rpm. Finally, sulfuric acid was transferred to an addition funnel and slowly dripped into the solution, maintaining stirring and keeping the temperature below 10 °C.

### Nitration Reaction

The preweighed cellulosic material was transferred into the round-bottom flask containing the prepared sulfonitric solution, under constant magnetic stirring at 500 rpm. The temperature was allowed to rise naturally and gradually to room temperature, and the flask was immersed in a silicone bath until the liquid level exceeded that of the reaction mixture. The system was gradually heated to 50 °C on the hot plate, maintaining an internal temperature between 30 and 35 °C, and stirred for 40 min. At the end of the reaction time, the flask was removed, and the reaction was quenched by immersion in an ice-water bath.

### Separation of the Reaction Mixture

After the reaction mixture was cooled to below 15 °C, vacuum filtration was performed using a porcelain Büchner funnel with a glass microfiber filter (GF/C, Whatman) attached to a side arm flask. A Jet Pump WEG motor (W56X210) was used to apply a vacuum. Subsequently, the solid product retained on the filter was washed and filtered with distilled water three times.

### Product Stabilization

The stabilization step of the nitrated product consisted of the following boiling processes: 1—in distilled water to remove excess acid and byproducts; 2—in an alkaline solution of sodium carbonate (Na_2_CO_3_) for neutralization; and 3—in distilled water to remove entrapped acidic and/or basic compounds.

The product was transferred to a two-neck round-bottom glass flask containing a magnetic stirrer, and distilled water was added until half of the volume was filled. The system was set up on a magnetic stirring hot plate (IKA model C-MAG HS 7) with a silicone oil bath. Under constant magnetic stirring at 500 rpm, it was gradually heated up to 150 °C on the hot plate, maintaining the internal temperature between 85 and 90 °C, and kept stirring for 90 min. At the end of the process, the flask was removed from the system and cooled by immersion in an ice-water bath. The product was then separated from the mixture by centrifugation using a NOVA Instrument benchtop centrifuge at 3000 rpm for 5 min, followed by removal of the aqueous phase (upper layer) with a Pasteur pipet.

Next, the entire process described was repeated using a Na_2_CO_3_ (0.3%) solution, which was added to the flask in a sufficient amount to neutralize the pH of the mixture. Finally, another boiling process with water was carried out under the same conditions as for the first. At the end of the stabilization step, the product was stored in a desiccator for complete drying at room temperature.

## Results and Discussion

This section presents the results of the analyses conducted to characterize the structural and thermal properties of the cellulosic materials used and their nitration products. It is worth noting that the following sections are organized to correspond to the specific objectives achieved during the development stages of this work.

First, the properties of the commercial celluloses provided for the study will be elucidated, including aqueous suspension CNF and cotton celluloses LC and MCC. Next, the main aspects of the changes in CNF caused by the different dehydration treatments are presented and discussed. Finally, the nitration experiments will be comparatively analyzed regarding the products obtained from CNF in relation to those from LC and MCC.

### Commercial Celluloses

#### Morphology

The surface morphology of the commercial cellulosic material was analyzed by using SEM images, shown in Figures 5 and 6.

**Figure 5:**
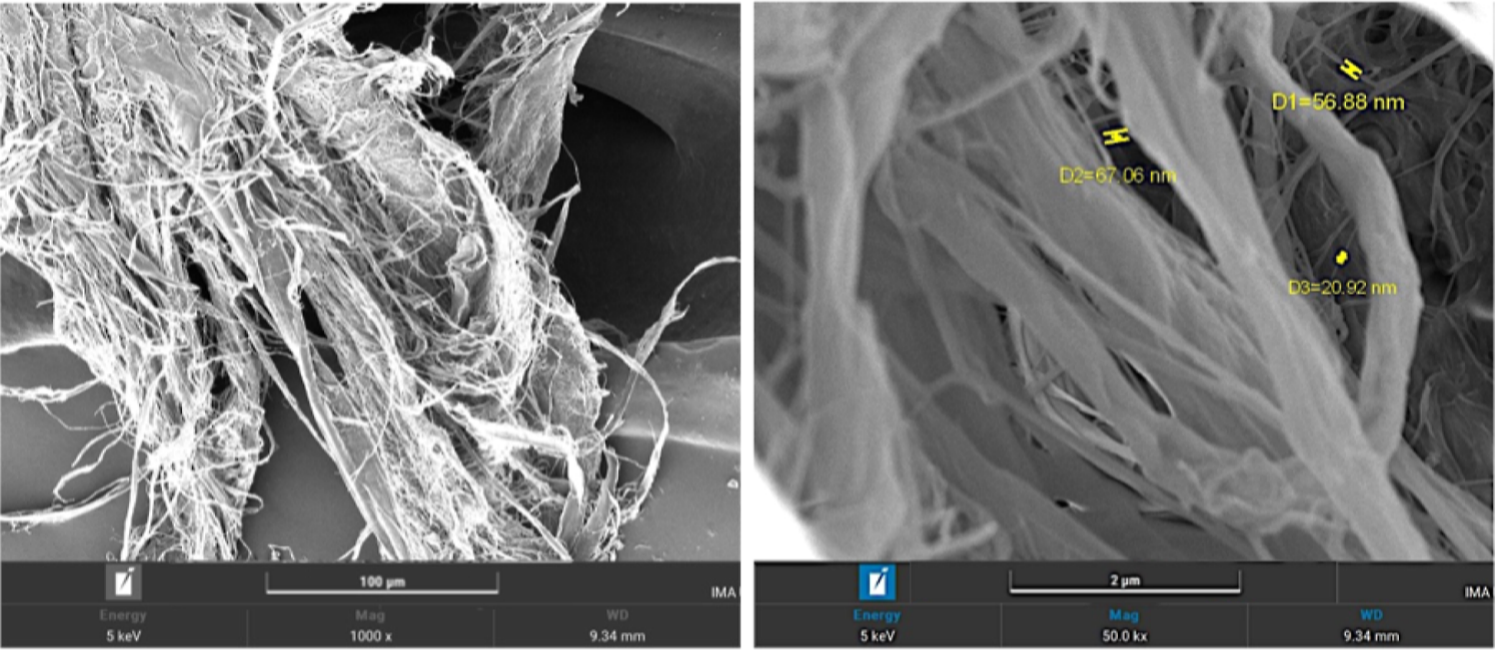
SEM images of the CNF sample at 1000× and 50,000× magnification.

**Figure 6:**
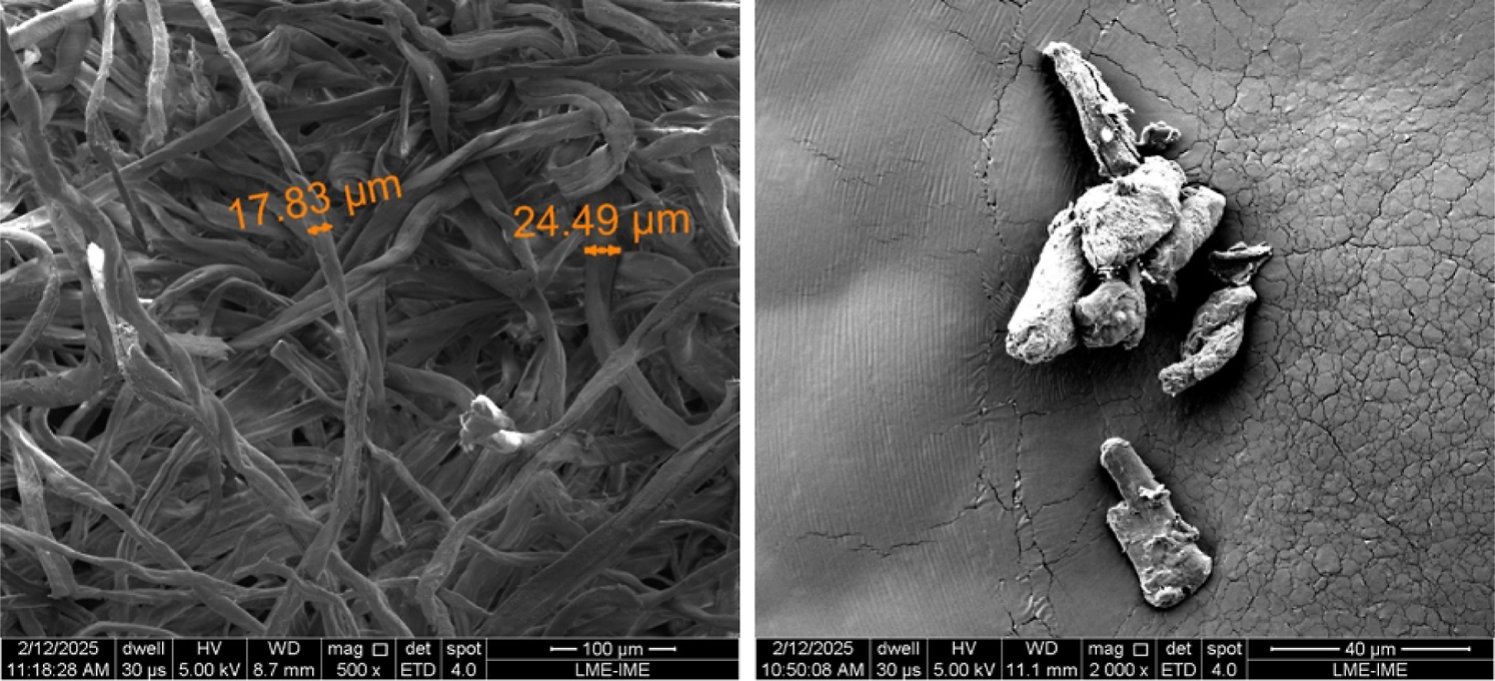
SEM images of the LC (left) and MCC (right) samples.

It is observed that the CNF predominantly exhibit a fibrillated shape with varied diameters. The smaller fractions measured in the 50,000× magnification image show nanometric dimensions, within the expected range for elementary fibrils, according to the literature. The fiber entanglements are a direct consequence of oven drying, which was necessary for the analysis. Indeed, water removal promotes the formation of a network through interfibrillar hydrogen bonds, favoring fibril agglomeration.^2^ LC displays long, flat, ribbon-like fibers with diameters in the micrometer range—15 to 25 μm—as expected for cotton-derived cellulose, according to the literature.^2,3^ MCC particles exhibited crystallite-like features with sizes close to 20 μm, consistent with their commercial specifications.

#### Molar Mass Distribution and Chemical Structure

SEC analyses provided the MMDs of the CNF and LC samples, presented as differential and cumulative mass fractions (WF) as a function of the logarithm of molar mass (MM) in Figure 7 and 8, respectively.

**Figure 7:**
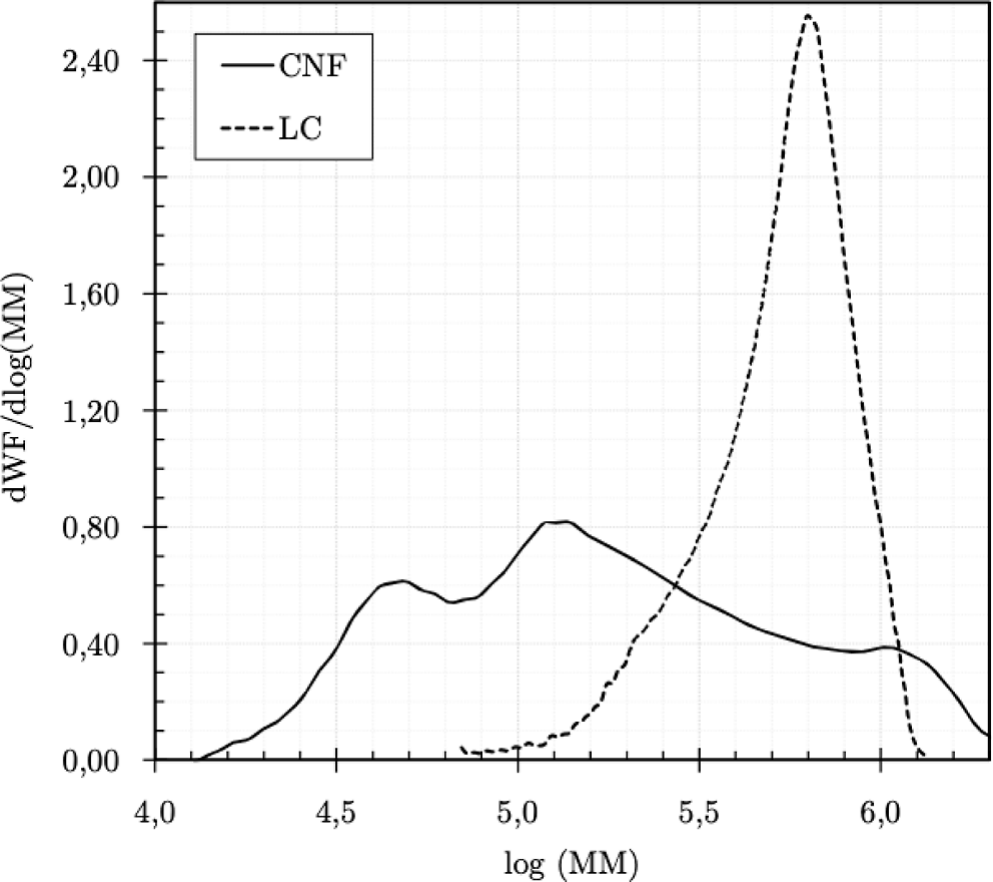
Differential MMD of the CNF and LC samples.

**Figure 8:**
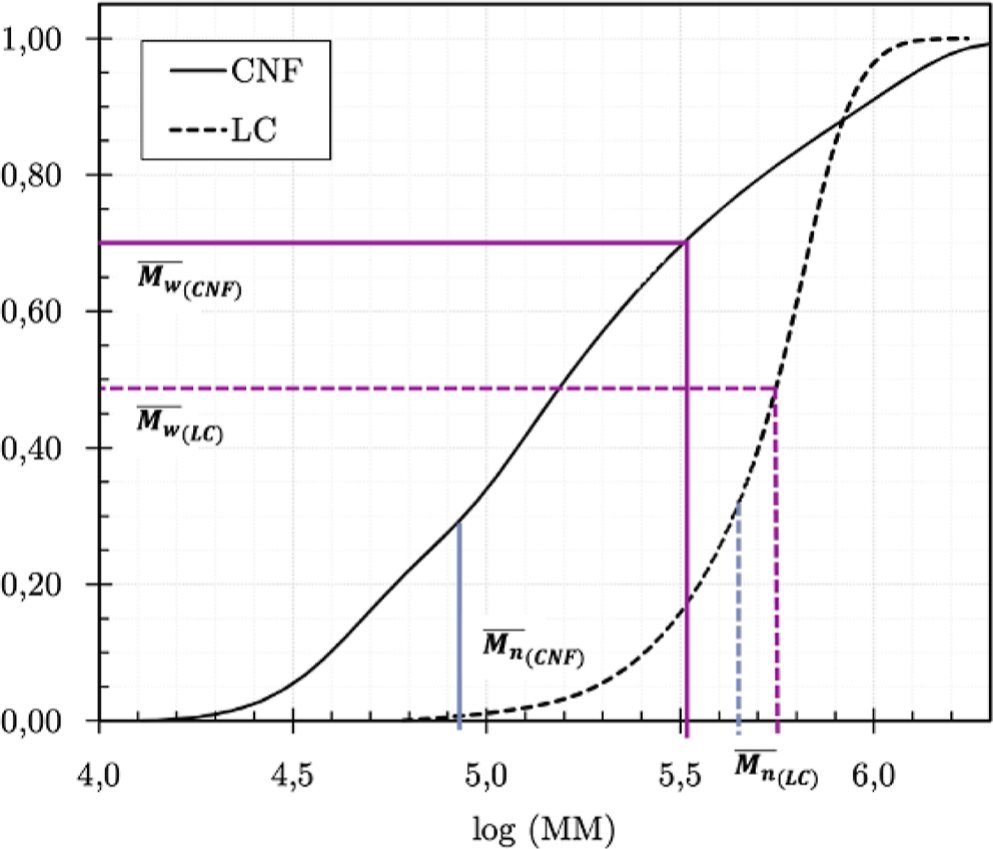
Cumulative MMD of the CNF and LC samples.

The differential MMDs reveal a noteworthy polydispersity in the CNF sample with polymeric fractions spanning chain length ranges comparable to those of the LC sample. This is due to the fact that CNF are usually produced by mechanical disintegration (e.g., homogenization, grinding), often combined with chemical pretreatments (e.g., TEMPO oxidation, enzymatic hydrolysis). These processes are not uniform and often cause random chain scission, leading to fragmentation and a range of molecular weights. The broad MMD of CNF arises from the natural variability in cellulose sources, the nonselective fragmentation during mechanical/chemical processing, and the heterogeneous morphology of the final material. Nevertheless, the molar mass of the CNF components is predominantly lower. The values calculated from the experimental data for *M*_n_, *M*_w_, and *D̵*, along with the arithmetic means and relative standard deviations (RSD), are listed in Table 2.

**Table 2:** Average Molar Masses and Dispersity Index (*D̵*) for CNF and LC Samples

parameter	measurement	CNF	LC
*M*_n_ (g/mol)	1	71,811	451,367
	2	98,775	434,899
	mean	85,293	433,133
	RSD (%)	22.35	2.63
*M*_w_ (g/mol)	1	334,425	568,051
	2	333,279	564,140
	mean	333,852	566,095.5
	RSD (%)	0.24	0.49
*D̵*	1	4.657	1.259
	2	3.374	1.297
	mean	4.0155	1.2780
	RSD (%)	22.59	2.10

Analyzing the results in Table 2 alongside the cumulative MMD, it is observed that the average *M*_w_ values $\left(\overset{®}{M_{w}\;}\right)$ —indicated by the dark lines in Figure 8—cover up to 70% of the polymeric fractions in CNF and about 50% of the LC sample. In both cases, this parameter was more representative than the average *M*_n_ values $\left(\overset{®}{M_{n}\;}\right)$ —indicated by the light lines in Figure 8—and was chosen for the SD calculation. Thus, using eq 2, the average numbers of AGU units were 2059 for CNF and 3491 for LC, with three times these values representing potential sites for nitration reactions.

FTIR–ATR spectroscopy allowed the investigation of the functionality of the cellulosic materials, as shown in the spectra in Figure 9.

**Figure 9:**
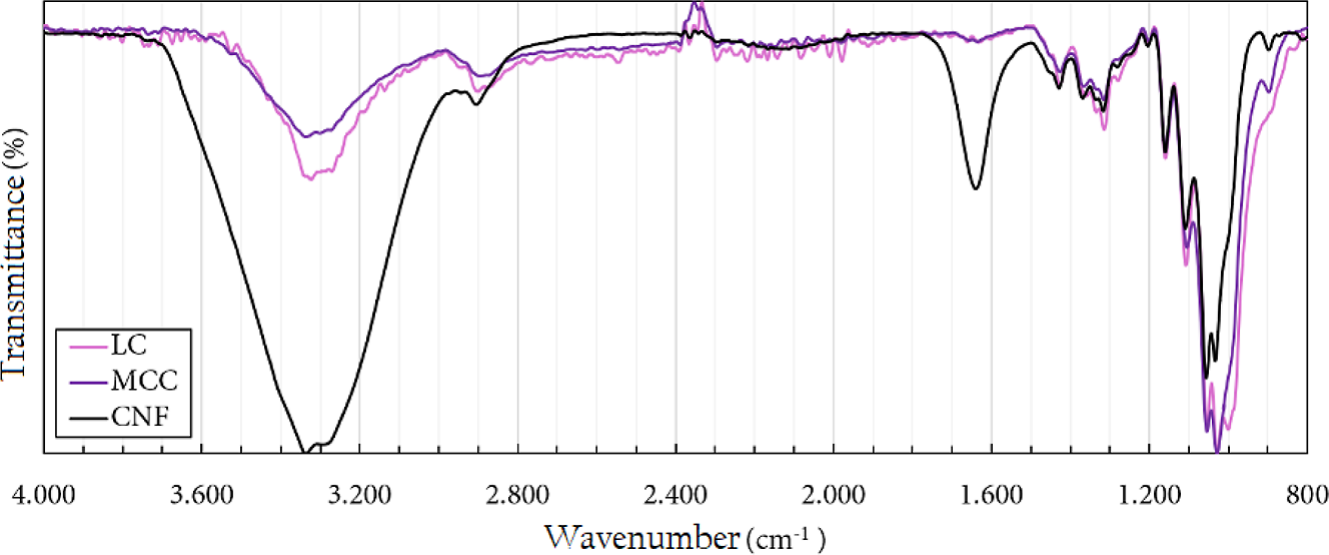
Spectra of the LC, MCC, and CNF samples.

The spectral profiles are consistent with the expected structural characteristics of a cellulose chain. The broad bands in the 3600 to 3000 cm^–1^ region correspond to the stretching vibrations of the −OH groups present in the AGU units, including covalent and intra- and intermolecular hydrogen bonds. It is worth noting that the spectra shown are from samples taken directly from their original packaging without pretreatment. Thus, CNF have a high water content—97% according to commercial specifications—which significantly contributes to the observed band profile and its higher intensity. The presence of water is also confirmed by the broad medium-intensity peak at 1640 cm^–1^, characteristic of the bending vibration of O–H bonds. The band centered near 2900 cm^–1^ is attributed to stretching vibrations, while the peaks at 1430 and 1315 cm^–1^ are related to the bending vibrations of C–H bonds. The region between 1200 and 950 cm^–1^ includes several peaks related to different C–O bond vibrations. The absorption peak at 898 cm^–1^ is attributed to the C–O–C stretching of the β-1,4-glycosidic bonds between AGU units (Luo et al., 2017).^100^

#### Thermal Behavior

Thermogravimetric (TG) and derivative thermogravimetric (DTG) analyses were used to investigate the thermal behavior of the commercial cellulosic material samples without pretreatment, as shown in the thermograms in Figures 10 and 11.

**Figure 10:**
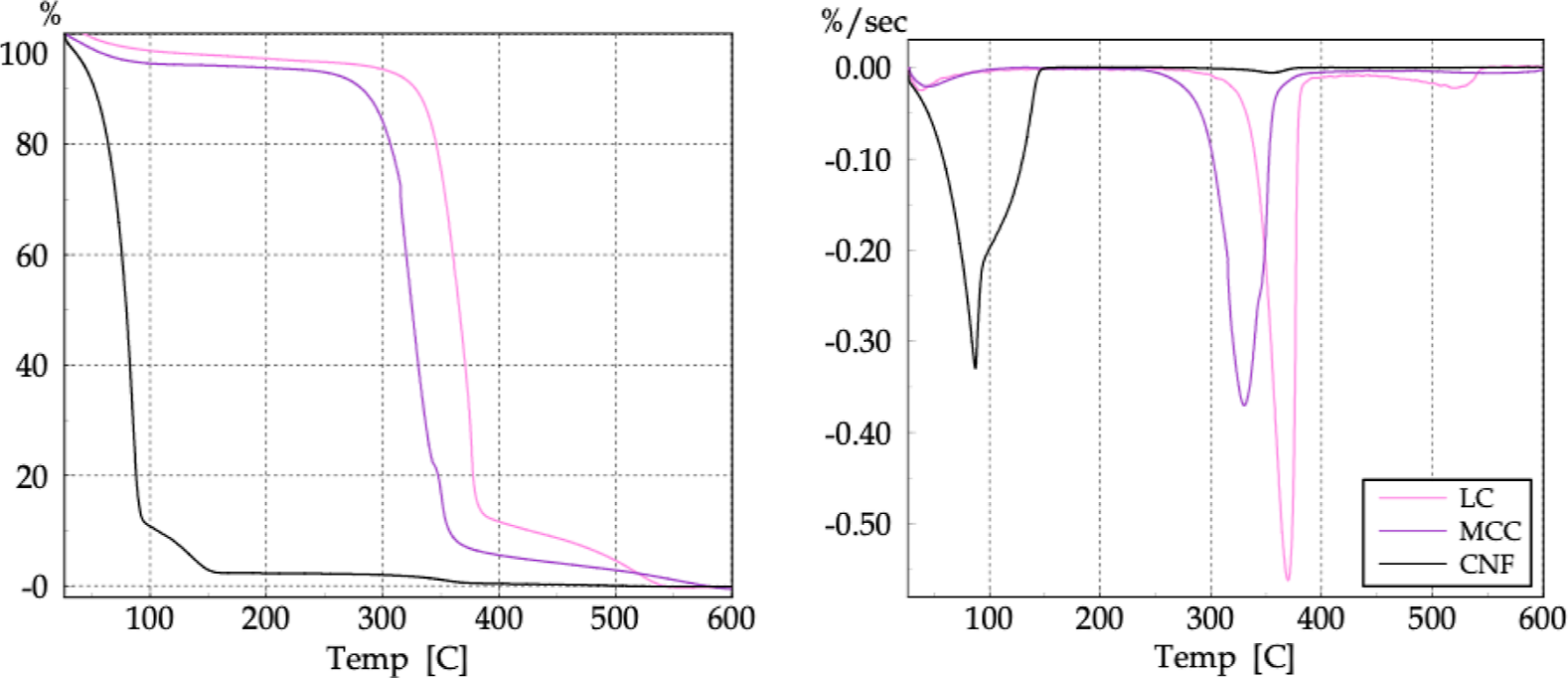
TG (left) and DTG (right) curves of the LC, MCC, and CNF samples.

**Figure 11:**
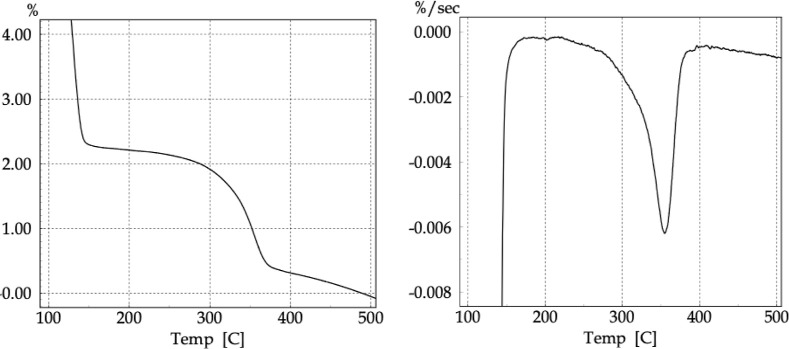
Enlarged TG (left) and DTG (right) curves of the CNF sample.

The three materials exhibited essentially three main events. The first is associated with the evaporation of adsorbed and surface-bound water. The LC and MCC samples showed a small mass loss of about 5%. However, the CNF analysis revealed a significant mass loss of 97.74%, corresponding to the water content of the sample, between 25 and 160 °C. This value is consistent with its commercial specification of a 3% solid content in aqueous suspension.

The thermal curves showed similar profiles regarding the main cellulose chain decomposition event associated with depolymerization through the cleavage of β-1,4-glycosidic bonds and AGU unit decomposition. Table 3 presents the mass loss Δ*m* (%) and initial (*T*_i_), final (*T*_f_), onset (*T*_onset_), and maximum degradation rate (*T*_max_) temperatures for this thermal event.

**Table 3:** TG and DTG Data Associated with the Main Thermal Degradation of the Original Cellulosic Material

sample	Δ*m* (%)	*T*_i_ (°C)	*T*_f_ (°C)	*T*_onset_ (°C)	*T*_max_ (°C)
LC	–82.88	273.46	400.00	350.35	369.79
MCC	–88.07	234.77	407.40	317.90	330.23
CNF	–1.85	223.95	393.67	324.71	354.85

The results showed that the decomposition of the samples occurs predominantly in one stage within the temperature range of 200 to 400 °C, consistent with the literature, and demonstrated that morphology affects thermal stability. This behavior depends mainly on the material preparation process. For LC and CNF, both fibrillated and containing crystalline and amorphous regions, it was observed that the decomposition temperatures decreased with dimensional reduction. The lower thermal stability of MCC may be due to partial degradation of the cellulose chains resulting from chemical treatment in its production process. The last decomposition event is attributed to the oxidation and decomposition of carbonized residues to form low-molecular-weight gaseous products.^39^

### Dehydrated CNF

The dehydrated CNF samples were initially characterized by TG/DTG to assess possible changes caused by the different dehydration treatments. The curves shown in Figure 12 allowed for a comparative investigation of the thermal behavior of the original and dehydrated CNF samples.

**Figure 12:**
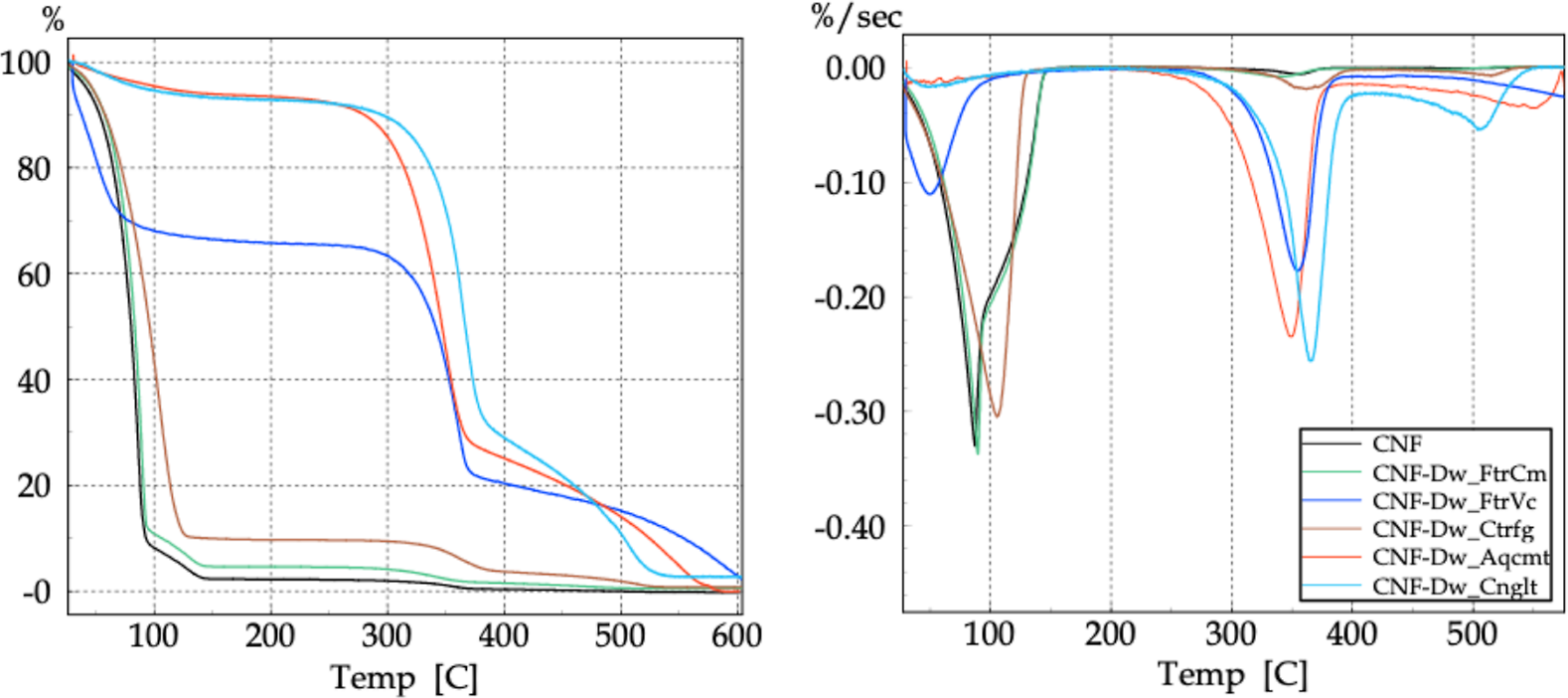
TG (left) and DTG (right) curves of the dehydrated CNF samples.

Overall, the profile of all curves shows the expected thermal events for cellulose, identical to those of the original CNF and MCC, indicating that the chemical structure of the material was not altered during the dehydration treatments. However, the thermal stability of the CNF is influenced by the different drying processes and the interactions between fibrils and water through hydrogen bonds, rather than changes in molecular bonds.

The CNF and CNF-Dw_FtrCm samples exhibited two stages related to the first thermal event. Due to the higher water content in these samples, it was possible to detect distinct mass loss processes corresponding to evaporation: first of “free” water forming a network around the fibrils, followed by “bound” water through hydrogen bonds directly with the −OH groups of the cellulose chains (Vyas et al., 2003).^101^ The water content was determined directly from the thermograms based on the characteristic mass loss profile of water (Vyas et al., 2003).^101^ The mass loss Δ*m* (%) and the *T*_onset_ and *T*_max_ temperatures of the thermal events associated with water evaporation and the main cellulose decomposition are presented in Table 4.

**Table 4:** TG and DTG Data Associated with Water Evaporation (Event 1) and the Main Thermal Degradation of Cellulose (Event 2)

	event 1			event 2		
sample	Δ*m* (%)	*T*_onset_ (°C)	*T*_max_ (°C)	Δ*m* (%)	*T*_onset_ (°C)	*T*_max_ (°C)
CNF	–97.74	71.77	87.44	–1.85	324.71	354.85
CNF-Dw_FtrCm	–95.42	78.81	89.76	–2.87	304.61	339.76
CNF-Dw_FtrVc	–29.17	41.57	51.32	–44.74	329.08	356.94
CNF-Dw_Ctrfg	–89.94	73.06	105.10	–5.98	333.31	362.18
CNF-Dw_Aqcmt	–6.02	-	-	–66.89	318.77	348.76
CNF-Dw_Cnglt	–6.83	34.64	46.49	–64.49	342.12	365.26

Common filtration and centrifugation removed only a small amount of water from the suspension, changing the fibril concentrations to 5% and 10%, respectively. Vacuum filtration concentrated the material to a 70% solid content. The heating and freezing methods were able to dry the CNF, reaching a moisture content similar to that observed for the LC and MCC samples.

Information about the crystalline structure of the dehydrated CNF was obtained using XRD. It was not possible to analyze the CNF and CNF-DwFtrCm samples due to their high “free” water content. Although the sample holder allowed proper placement of both, water leakage was observed when positioning it vertically for insertion into the equipment. Figure 13 shows the diffractograms of the analyzed samples. The diffraction profiles exhibited the typical cellulose pattern, with characteristic peaks associated with the (1 −1 0), (1 1 0), (2 0 0), and (0 0 4) crystallographic planes, as shown.

**Figure 13:**
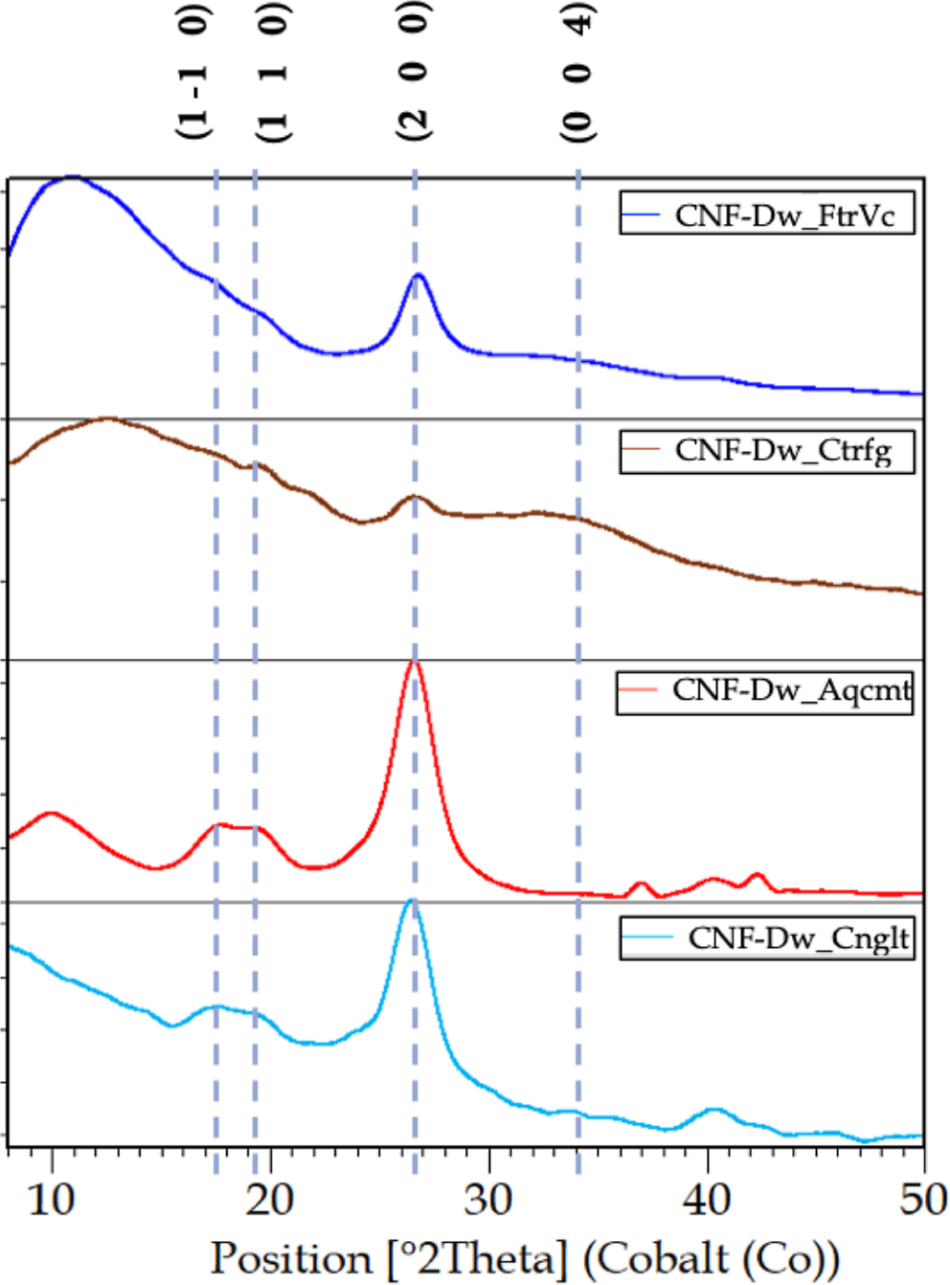
Diffractograms of the dehydrated CNF samples.

The diffraction profiles show overlapping peaks, forming broad bands, characteristic of the presence of an amorphous portion in the material. Additionally, XRD requires a minimum crystallite size to consider a fraction as crystalline. CNF have nanoscale dimensions maintained by dispersion, in this case, in an aqueous phase. Thus, samples with higher water content exhibit broader crystalline peaks with lower relative intensity, as seen in the diffractograms of CNF-Dw_FtrVc and CNF-Dw_Ctrfg. In other words, a pattern with lower peak intensities is not necessarily less crystalline.

The CrI of the samples calculated using the Segal method is presented in Table 5. Analyzing the results, a correlation between the water content of the samples and the crystallinity index is observed, with the reduction evidenced in the diffractograms by a decreased intensity and increased width at half-height of the diffraction peaks.

**Table 5:** Crystallinity Index of the Dehydrated CNF Samples

sample	% *m*_w_ (TGA)	CrI_Segal_ (%) (XRD)
CNF-Dw_FtrVc	29.17	54.6
CNF-Dw_Ctrfg	89.94	15.3
CNF-Dw_Aqcmt	6.02	85.6
CNF-Dw_Cnglt	6.83	59.4

The Segal height method provided a simplified way to evaluate changes in the crystalline content of CNF after the treatments used to concentrate the fibrils. Being empirical, its development was aimed at quantifying differences among a set of samples from the same source, as in this case. However, some limitations should be noted. The method assumes that all intensity at the minimum between the peaks related to the (1 1 0) and (2 0 0) planes is from amorphous scattering, disregarding the overlap of broad peaks resulting from smaller crystallites.

The FTIR–ATR spectra, shown in Figure 14, provided a complementary method to confirm the structural characteristics of the dehydrated CNF and the observations made via XRD.

**Figure 14:**
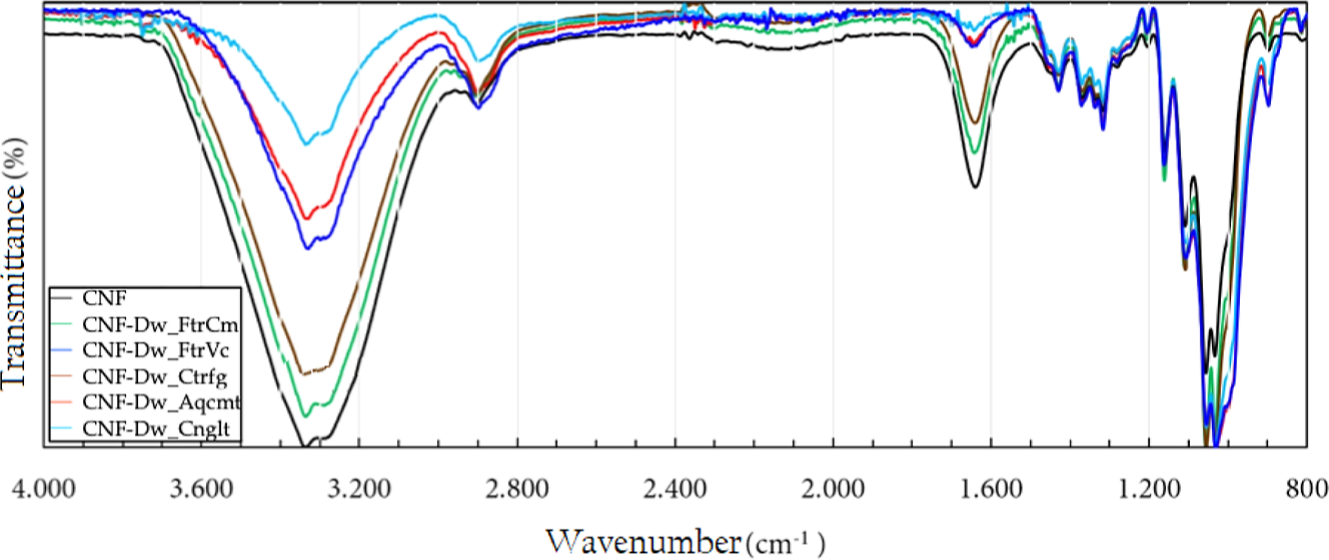
Spectra of the original and dehydrated CNF samples.

The results indicate that the initial and onset temperatures of decomposition varied, depending on the dehydration method. However, no apparent differences in the spectral profiles of the samples were discernible—the same number of decomposition events was observed—indicating that no chemical reactions occurred and/or no new substances were produced during the different treatments. Thus, the observed differences in thermal stability can be attributed to the morphology of the nanostructure after the treatments, particularly for those that are more efficient in terms of water removal.

As indicated by Manimaran et al.,^40^ a positive correlation has been established between crystallinity and thermal stability. Although oven-dried CNF exhibited a significantly higher crystallinity index (85.6%), their lower *T*_onset_ (318.77 °C) compared to freeze-dried CNF (342.12 °C, with 59.4% CrI) indicates that crystallinity alone does not exclusively dictate the thermal stability of nanocellulosic systems.^41^ Similar results were reported by Sharma et al., using different oven temperatures to dry nanocellulose.^42^ Freeze-drying is typically a slow water-removal process that produces a structural morphology consisting of a dense solid top surface and a porous, sponge-like bulk structure.^43^ Consequently, the initial decomposition temperature is observed to be marginally lower; however, as the temperature rises, an acceleration in the degradation process becomes evident due to the increased surface area of exposure, as supported by the experimental findings. Additionally, the freeze-drying process preserves the fibrillar network and minimizes hornification and thermally induced defects, resulting in a nanoarchitecture that delays the onset of depolymerization despite a higher amorphous content.^41^ As for the other drying treatments, the experimental trend was greater stability for a higher crystallinity index of the CNF structure.

As discussed earlier, the broad bands in the 3600 to 3000 cm^–1^ region are associated with stretching vibrations of –OH groups, including intra- and intermolecular hydrogen bonds. The analyzed samples contain water in their composition, the content of which directly and proportionally influences the band intensity, as observed in the spectra and also by the broad medium-intensity peak at 1640 cm^–1^, characteristic of the bending of adsorbed water O–H bonds. The absorption peaks at 1430 and 898 cm^–1^—attributed to the asymmetric bending vibration of C–H bonds and the C–O–C stretching of the β-1,4-glycosidic bonds between AGU units, respectively—are related to the material’s crystallinity. The first represents the “crystalline band,” while the second represents the “amorphous band” (Luo et al., 2017; Salem et al., 2023).^100^ The observed changes in relative intensities confirm the results obtained via XRD, showing that CrI decreases as the intensity of the first absorption decreases, while the intensity of the second increases.

### Nitration Products

The synthesis of NC from CNF was possible only with the sample dehydrated by vacuum filtration. LC and MCC were nitrated under identical conditions for comparative purposes. Attempts to nitrate CNF by centrifugation were made, but no product was obtained during separation of the reaction mixture. A possible explanation for this would be the complete decomposition of the cellulosic material by the acids. CNF dried by heating and freezing were not tested as it was not possible to redisperse them after the respective treatments.

The marked dependence of nitration feasibility on the dehydration method applied to CNF can be rationalized in terms of well-established structural phenomena in cellulosic materials, particularly hornification, nanofibrillar network collapse, and loss of chemical accessibility. During drying or severe consolidation processes, such as heating, freeze-drying, and centrifugation, cellulose nanofibrils undergo irreversible rearrangement driven by the formation of extensive interfibrillar hydrogen bonding. This process, commonly referred to as hornification, leads to pore collapse, densification of the nanofibrillar network, and a substantial reduction in accessible surface area.^44–46^

Previous studies have demonstrated that even when the primary chemical structure of cellulose remains unchanged, these physical transformations result in loss of redispersibility and severely limit the accessibility of hydroxyl groups, which are essential reactive sites for heterogeneous chemical modifications.^47,48^ In the context of nitration, such densification hinders the diffusion of nitrating species into the cellulose matrix and promotes nonuniform acid penetration, which may favor hydrolytic degradation or chain scission over esterification.

In contrast, partial dehydration by vacuum filtration primarily removes free and weakly bound water while preserving the hydrated nanofibrillar architecture and maintaining interfibrillar spacing. This approach minimizes hornification and sustains chemical accessibility, thereby enabling effective interaction between the nitrating agents and the cellulose hydroxyl groups. Similar sensitivity of nanocellulose reactivity to physical consolidation has been reported for other heterogeneous chemical modification routes, such as TEMPO-mediated oxidation, where excessive densification or collapse of the nanofibrillar network results in reduced reactivity or degradation of the cellulose chains.^49^ Consequently, the present observations are consistent with the broader literature on nanocellulose processing and underscore the critical role of dehydration-induced structural effects in determining the feasibility of the nitration reactions.

The obtained products were identified by FTIR–ATR. The spectra of the starting materials (dashed lines) and nitration products (solid lines) of LC, MCC, and CNF are shown in Figure 15. In the high-frequency region, the two broad medium-intensity bands around 2890 and 3340 cm^–1^ were again observed, originating from the stretching vibrations of the C–H bonds and the remaining –OH groups in the AGU units, respectively. A comparison between the spectra of the starting materials and nitration products shows reduced relative intensities, indicating partial substitution of hydroxyl groups.

**Figure 15:**
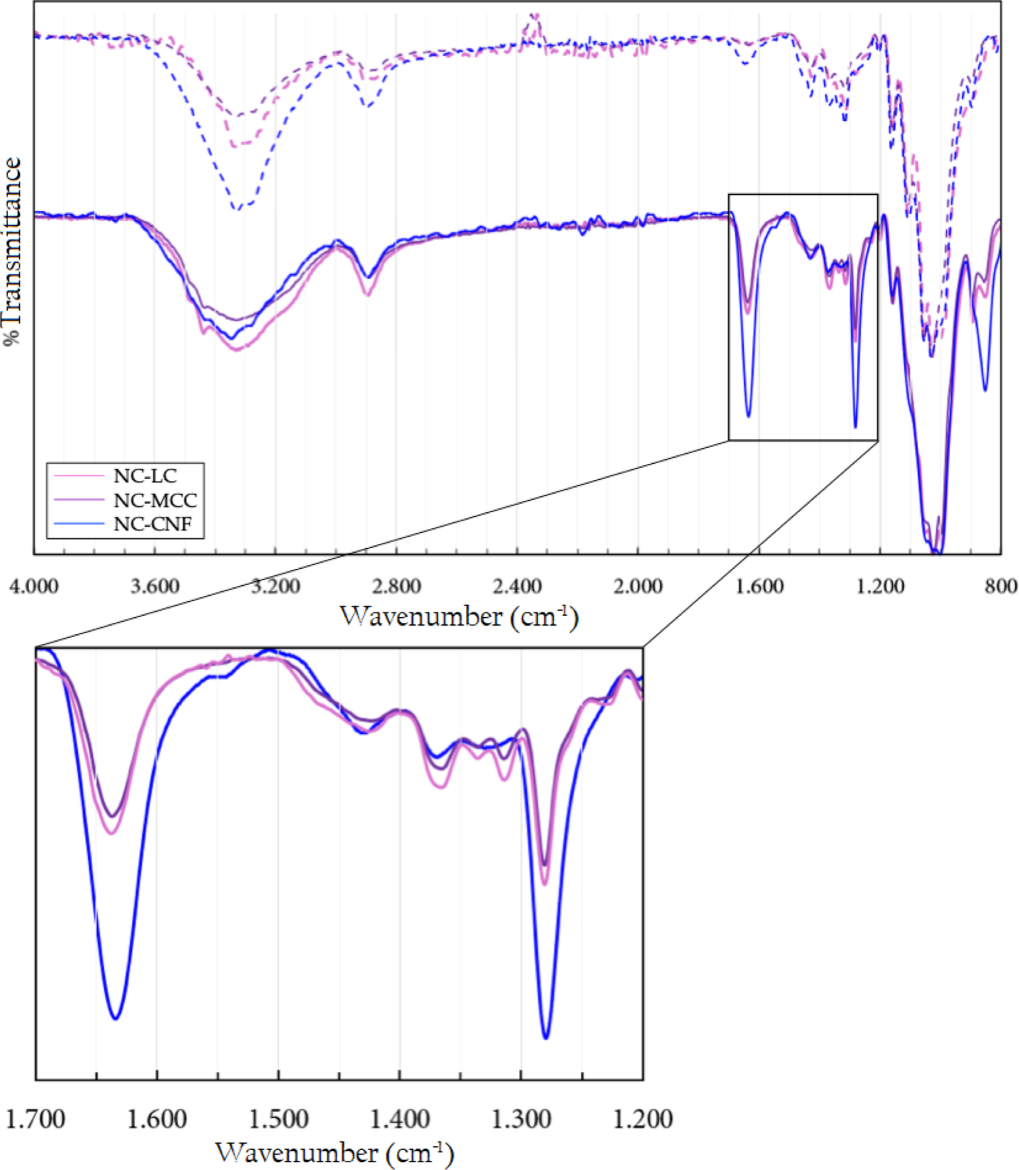
Spectra of the starting materials (dashed) and nitration products (solid) of LC, MCC, and CNF.

After nitration, the spectra exhibit the characteristic absorption pattern of the nitrate ester functional group, confirming the reaction. The sharper and more intense peaks at 1280 and 1650 cm^–1^ correspond to the symmetric and asymmetric stretching of the –NO_2_ groups, respectively. There is also a slightly broader intense peak around 832 cm^–1^ attributed to the stretching of the O–NO_2_ bond.^36^

When comparing the spectra of the products obtained from the different cellulosic materials, the intensities of these peaks—related to the –NO_2_ groups—were similar for LC and MCC but increased for CNF, suggesting that the smaller dimensional scale characteristic of the material favors the production of a more nitrogenated product. Indeed, this observation was confirmed by elemental analysis results, where the nitrogen content allowed evaluation of the degree of substitution (*z*) using eq 7, as shown in Table 6.

**Table 6:** Nitrogen Content and Degree of Substitution of the Nitrated Products

starting material	% N	*z*
LC	3.65	0.71
MCC	3.72	0.75
CNF	8.98	1.79

Increasing the reaction time, the concentration of the acids involved, and the acid-to-cellulose ratio could be used to raise the nitrogen content. However, since such adjustments could, among other detrimental effects, promote parasitic oxidation reactions, fiber degradation, or even ignition of the reaction mixture, we chose to maintain reaction conditions representative of those commonly employed in industrial environments.

Despite the low degrees of substitution achieved under the experimental reaction conditions, it is worth noting that an inverse relationship between nitrogen content and the dimensions of the starting materials may be suggested, since a larger specific surface area is generally associated with increased availability of reactive sites. Thus, it would be expected that the nitrogen content would increase with dimensional reduction. The NC-CNF obtained with *z* ≈ 2 means that two nitrate esters were formed per cellobiose, assuming homogeneous distribution. The result for NC-MCC may be associated with the physicochemical properties of MCC. In this case, the predominance of crystalline regions leads to tighter crystal packing, hindering the diffusion of nitrating species into the polymer matrix and reducing the accessibility to hydroxyl groups.

Figures 16 and 18 show the SEM images of the different cellulose samples subjected to nitration and their respective products.

**Figure 16:**
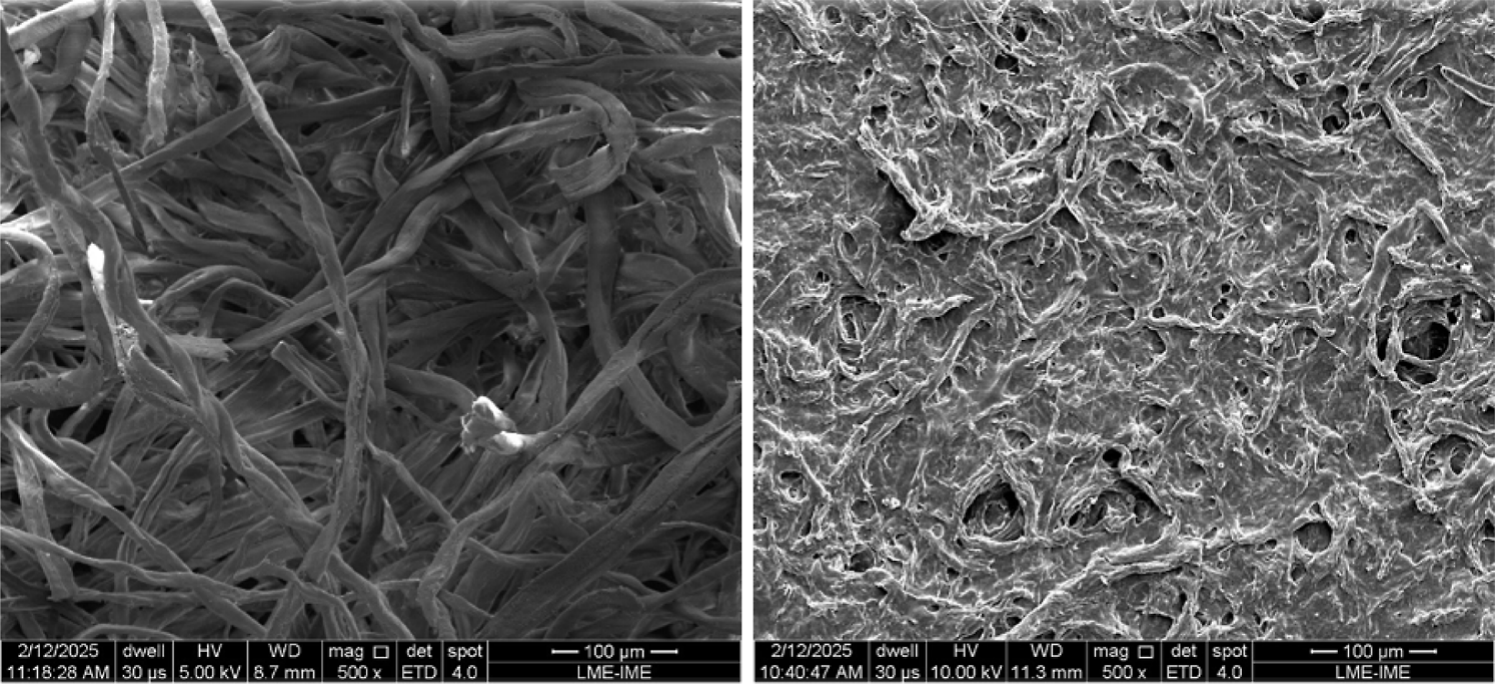
SEM images of the LC (left) and NC-LC (right) samples.

**17:**
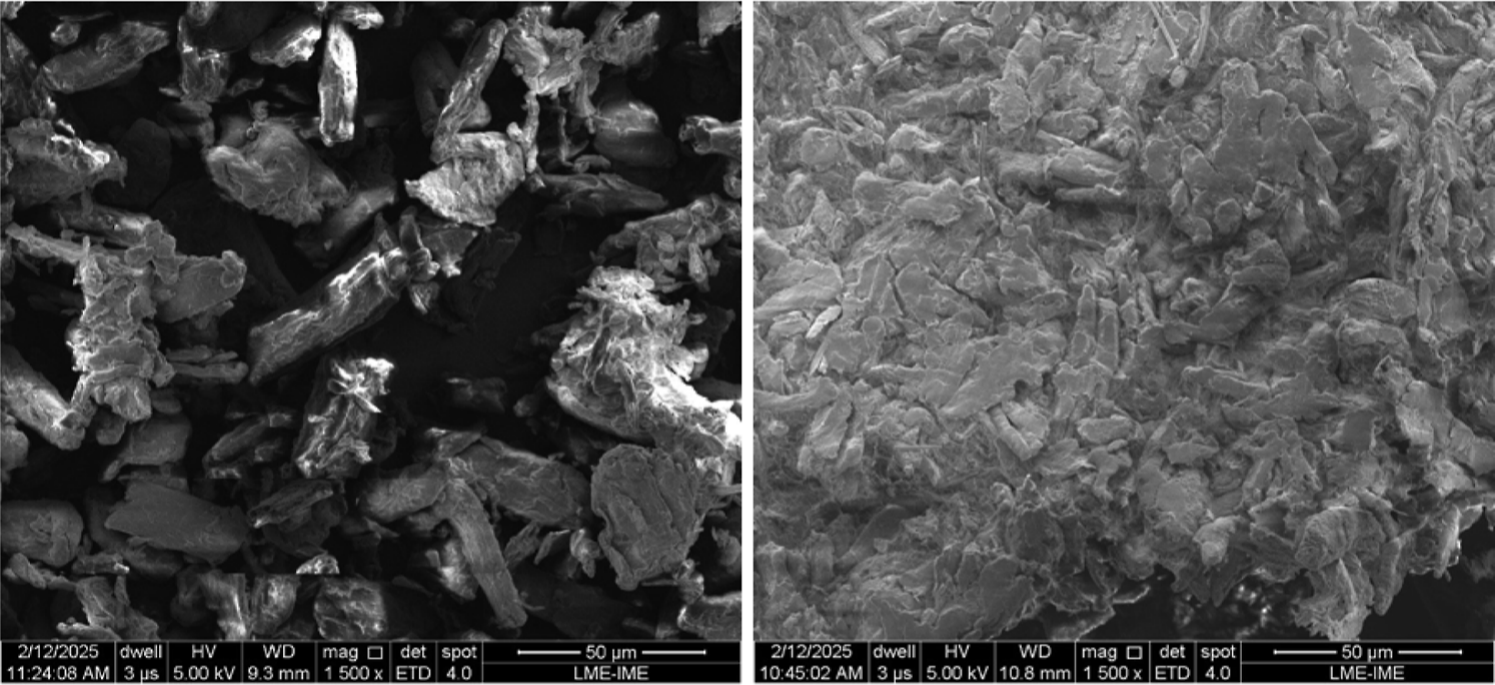
SEM images of the MCC (left) and NC-MCC (right) samples.

**Figure 18:**
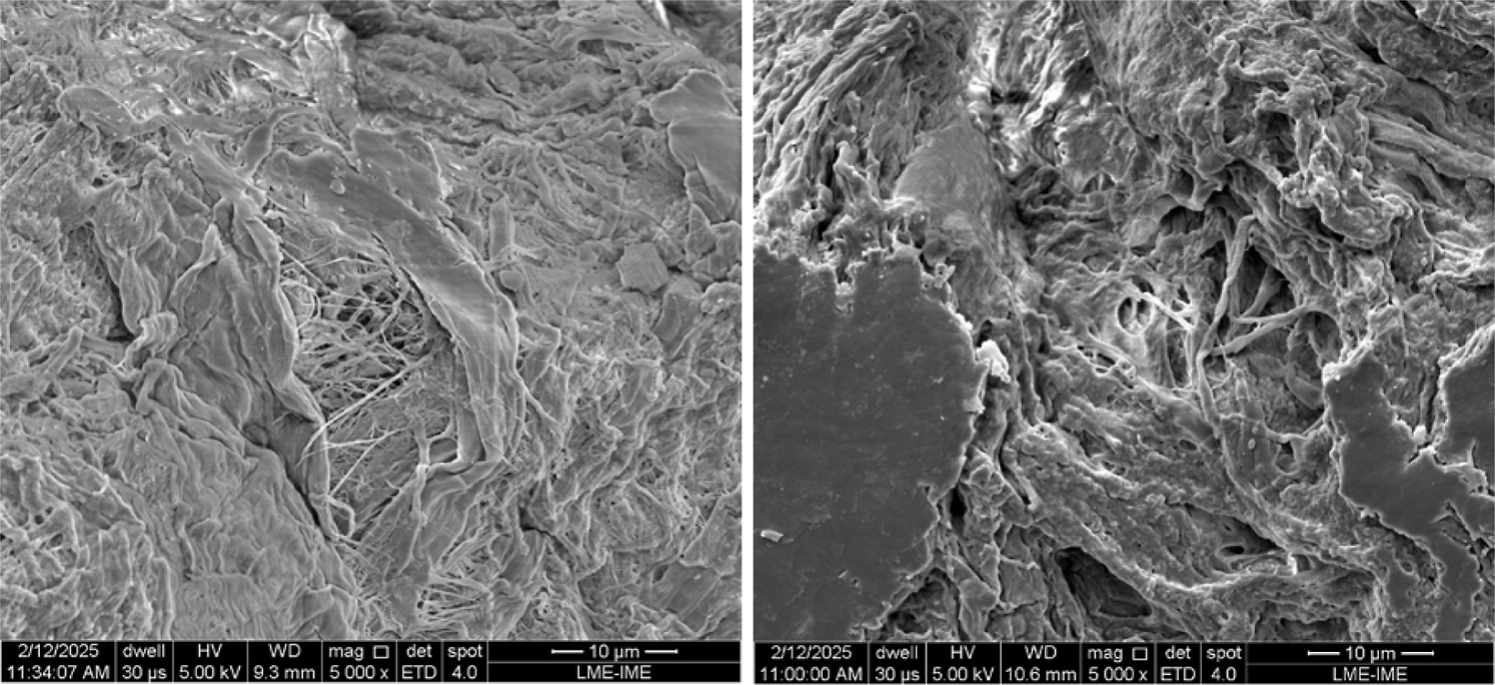
SEM images of the CNF-Dw_FtrVc (left) and NC-CNF (right) samples.

The SEM images (Figures 16–18) reveal that nitration had distinct effects on fibril morphology, depending on the cellulose precursor. For LC and MCC, the nitrated products (NC-LC and NC-MCC) exhibited partial fragmentation of the original micrometer-sized fibers and crystallite-like particles, suggesting that the sulfonitric medium promoted surface erosion and dimensional reduction. In contrast, NC-CNF preserved its fibrillated network appearance, although the bundles appear less compact and more fragmented compared to the starting CNF, which indicates partial disruption of interfibrillar hydrogen bonds during nitration. This observation is consistent with the higher nitrogen incorporation measured for CNF (Table 6), since the increased accessibility of hydroxyl groups along thinner fibrils favors substitution. The visible interface lines reveal the fibrous structure of NC-LC and NC-CNF, as well as the crystallites of NC-MCC. It was not possible to measure the diametrical dimensions of the NC due to agglomeration. Additionally, interaction between the nitrated materials and the microscope’s electron beam caused successive changes in the images during capture, making focusing difficult and resulting in loss of sharpness. However, an apparent dimensional reduction was observed for LC and MCC. NC-CNF exhibited a network structure with fiber diameters comparable to CNF.

The change in the crystalline structure of the cellulosic material after nitration can be observed in the diffractograms shown in Figure 19. The typical cellulose peaks—associated with the (1 −1 0), (1 1 0), (2 0 0), and (0 0 4) crystallographic planes—are highlighted and indicate that the NC has a diffraction profile with distinct characteristics. Nitration notably promoted a reduction in crystallinity between the raw material and product, as evidenced by the broadening of the diffraction peaks. This occurs due to fewer interactions via hydrogen bonds, as some –OH groups were replaced by –NO_2_ groups.

**Figure 19:**
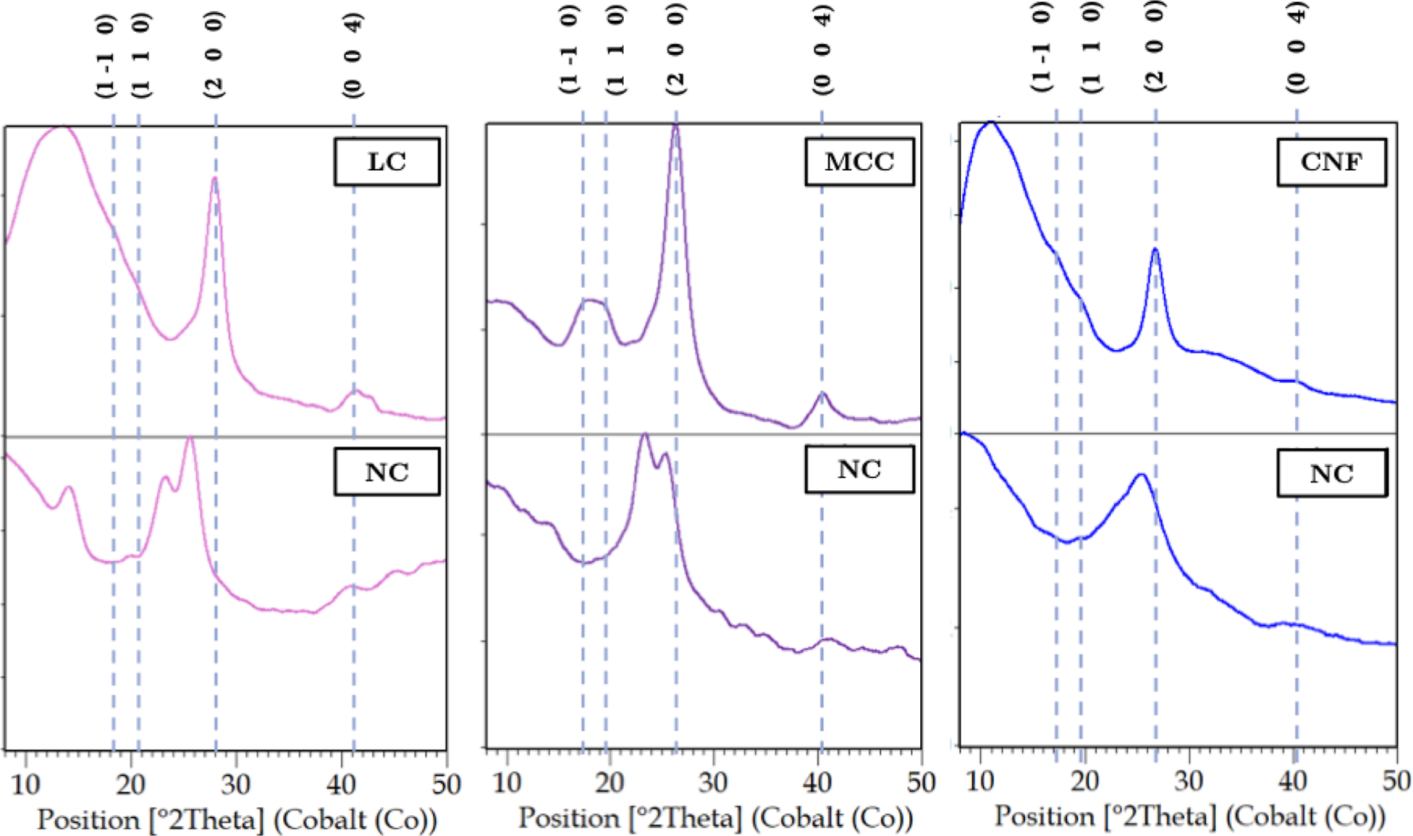
Diffractograms of the starting materials (top) and nitration products (bottom) of LC, MCC, and CNF.

TG/DTG analyses were used to investigate the thermal behavior of the synthesized NC, as shown in the curves in Figure 20. Visually, the thermal curves showed similar profiles with regard to the decomposition events.

**Figure 20:**
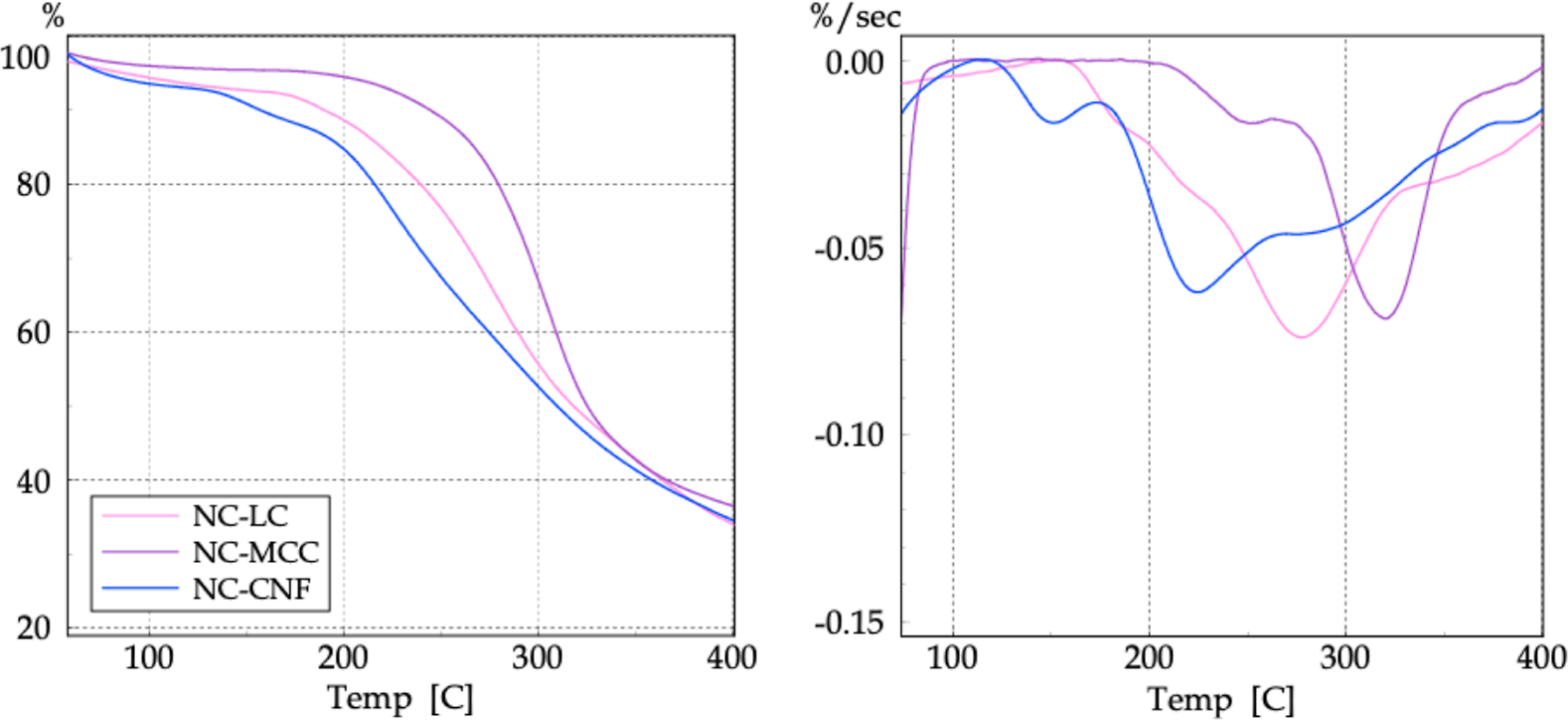
TG (left) and DTG (right) curves of NC.

The thermal decomposition of nitrocellulose (NC) is strongly influenced by the structure of parent cellulose, as differences in size scale, crystallinity, surface chemistry, and supramolecular organization lead to distinct decomposition mechanisms and kinetics.^50^ Due to the low nitrogen content achieved, the TG curves did not show a single mass loss process with a narrow DTG peak, characteristic of nitrocellulose as reported in the literature.^51^ The insertion of –NO_2_ groups reduces the thermal stability of cellulosic materials, as the activation energy for the decomposition of N–O bonds is lower than that of C–O bonds between AGU units. Thus, cellulose decomposition occurs first in chains with a higher nitrate ester content, while at higher temperatures, chains with lower degrees of substitution decompose. Comparing the *T*_onset_ values of the second stage, which showed a more pronounced mass loss for all samples, it is clear that morphology indeed affects thermal stability. NC-LC and NC-CNF exhibited thermal behavior patterns similar to their respective starting materials, i.e., decomposition temperatures decreased with dimensional reduction.^9^ Such behavior was also confirmed by Dobrynin et al.^14^ and Tarchoun et al.^52^ This was not the case for NC-MCC, as its predominantly crystalline structure contributed to an increased thermal stability. The first stage may also be associated with gradual mass loss due to water evaporation or even low-weight compounds retained in deeper fiber layers. In all samples, a small initial mass loss associated with surface-adhered water evaporation was also observed.

Due to the low nitrogen content achieved, the TG curves did not show a single pronounced exothermic behavior with a narrow exothermic peak, characteristic of nitrocellulose as reported in the literature. The insertion of –NO_2_ groups reduces the thermal stability of cellulosic materials, as the activation energy for the decomposition of N- and O-bonds is lower than that of C- and O-bonds between AGU units. Thus, cellulose decomposition occurs first in chains with higher nitrate ester content, while at higher temperatures, chains with lower degrees of substitution decompose.

The results revealed that the main thermal degradation of the obtained NC occurred in two stages, with the temperatures *T*_i_, *T*_f_, *T*_onset_, and *T*_max_ listed in Table 7.

**Table 7:** TG and DTG Data Associated with the Main Thermal Degradation of NC

	stage 1				stage 2			
sample	*T*_i_	*T*_f_	*T*_onset_	*T*_max_	*T*_i_	*T*_f_	*T*_onset_	*T*_max_
NC-LC	192.55	206.84	206.84	-	223.54	336.59	251.23	274.80
NC-MCC	209.49	266.23	229.97	253.99	266.23	360.99	296.32	320.32
NC-CNF	116.78	174.61	133.45	151.46	174.61	277.14	199.83	224.22

Comparing the *T*_onset_ values of the second stage, which exhibited a more pronounced mass loss for all samples, suggests that morphology may influence the apparent thermal stability. NC-LC and NC-CNF exhibited thermal behavior patterns similar to their respective starting materials; i.e., decomposition temperatures decreased with dimensional reduction. This was not the case for NC-MCC, as its predominantly crystalline structure contributed to increased thermal stability.

The first stage may also be associated with gradual mass loss due to water evaporation or even low-weight compounds retained in deeper fiber layers. In all samples, a small initial mass loss associated with surface-adhered water evaporation was also observed.

The thermal analysis results provide important insights when they are evaluated against the performance requirements of nitrocellulose in energetic applications. Industrially, nitrocellulose used in propellants and explosives typically exhibits an onset of decomposition in the range of 180–200 °C, with higher nitrogen content generally correlating with lower stability but higher energetic output. In this study, the nitrated products (NC-LC, NC-MCC, and NC-CNF) displayed decomposition profiles with onset temperatures below 230 °C (Table 7), consistent with partially nitrated cellulose derivatives.

For practical relevance, these results indicate that while the obtained degrees of substitution are not yet sufficient for high-performance propellant-grade NC (which requires nitrogen contents above 12.5% and stable decomposition profiles above 180 °C), the observed reduction in onset temperature with decreasing particle size (notably in NC-CNF) aligns with literature reports linking nanoscale morphology to increased reactivity. This suggests that CNF-derived nitrocellulose, once optimized to reach higher nitrogen incorporation, could exhibit enhanced combustion efficiency and faster energy release, features desirable for advanced energetic formulations.

Therefore, even though the current products fall short of industrial specifications, the trends identified—namely, the morphology-dependent thermal stability and decomposition kinetics—demonstrate the potential of nanostructured precursors as a route to tailor the balance between stability and reactivity required for next-generation energetic materials.

## Conclusions

The main objective of this work was to develop a methodology for synthesizing nitrocellulose from nanostructured celluloses, commercially available as nanofibers in aqueous suspension, aiming at the production of materials applicable as energetic compounds. To achieve this goal, dehydration methods were investigated to concentrate the nanofibers in suspension, and nitration experiments were conducted with dehydrated CNF to define procedures and reaction conditions for product formation.

This study demonstrated the potential feasibility of the synthesis methodology developed, based on consistent findings in the literature regarding the nitration of celluloses of various origins, dimensions, and morphological types. Although the resulting degree of substitution was not adequate for energetic applications, it was possible to obtain nitrated products from the commercial cellulosic materials under study, indicating the viability of the employed processes.

The investigation of partial dehydration methods for the cellulose nanofiber suspension revealed that the chemical structure of the cellulose chains is not altered by water removal or by the temperature and pressure conditions applied. However, fibril agglomeration is favored due to the interconversion of hydrogen bonds between cellulose and water and cellulose and cellulose, making the redispersion of nanofibers difficult or even unfeasible, as observed in the heating and freezing treatments.

Only vacuum filtration proved to be suitable for preparing CNF for the reaction and obtaining nitrocellulose. The advantage of this method lies in its nondestructive and scalable nature, making it the most promising for use as a preparatory step in the nitration protocol.

The procedures and reaction conditions established for obtaining nitration products from concentrated cellulose nanofibers are not yet sufficient for propellant-grade nitrocellulose applications but are nevertheless promising, as evidenced by the significantly higher nitrogen content achieved for CNF (8.98%) compared to conventional cotton-based celluloses under identical conditions. This value lies at the threshold of the economically and technologically relevant range for energetic nitrocellulose derivatives, highlighting the intrinsic reactivity advantage conferred by the nanostructured morphology. Accordingly, the present work is inherently prospective, demonstrating that CNF constitute a promising feedstock for the development of energetic polymers. Further optimization of key process parameters—such as the composition of the nitrating agent, the nitrating agent-to-cellulose ratio, and the reaction time and temperature—is expected to enable higher degrees of substitution while preserving the favorable structural characteristics of cellulose nanofibers.

By comparison of the structural and thermal properties of the nitrated cellulose nanofibers with those of nitration products from conventional commercial cotton-based celluloses obtained under identical conditions, the results were consistent with findings in the literature and established theories. These findings align with the interests of the energetic materials field in exploring nanocellulose derivatives as potential substitutes for traditional ingredients.

Although SEM provides qualitative insights, an accurate assessment of morphological changes would benefit from a particle size distribution analysis. Annotated images highlighting fibril thinning and bundle fragmentation or image analysis using software to quantify diameter distributions could strengthen these observations. Therefore, TEM imaging is suggested to directly visualize the nanoscale changes in fibril integrity and assess whether nitration induces local defects or preserves the crystalline domains of the CNF. Such complementary data would provide a more comprehensive correlation between morphology and the efficiency of nitrogen incorporation in different cellulose substrates.

The development and improvement of energetic materials have been the subject of intense scientific research within the Brazilian Army. There are experimental efforts focused on technological alternatives for the production of next-generation propellants. In this context, this work contributes to this effort by aligning with the initiative of nitrating commercial nanofibrillated celluloses to obtain products that offer advantages in physicochemical, thermal, and mechanical properties, as well as optimized reaction parameters.

Some additional studies directly related to this work have proven to be of interest. The dehydration methods for CNF could have their execution conditions optimized and expanded to explore other alternatives such as water removal through fluidized bed drying. The characterization of the crystalline structure of the dehydrated materials as well as of the nitration products could be investigated in more depth, with efforts directed at calculating the crystallinity index through different methodologies. Other important parameters to study include the moisture content and viscosity of the resulting NC, as they may influence the burning rate in ballistic applications.

There are also further future perspectives arising from this research line, such as the study of blends of nanocellulose nitrates with conventional NC for applications with more demanding operational requirements, such as in the aerospace industry. It is expected that the gelatinization properties of the obtained material, particularly the mechanical properties, will remain stable for longer periods than those of the traditional substitute, allowing its use in molded propellant grains.

In a strategic synthesis of the results obtained, this study presents a systematic comparison between nanofibrillated cellulose and conventional industrial celluloses subjected to identical sulfonitric nitration conditions with emphasis on the role of precursor morphology and consolidation history. In contrast to previous studies that primarily address postnitration size reduction, composite formulations, or isolated nanocellulose systems, the results indicate that the preservation of nanoscale accessibility prior to nitration influences both nitrogen incorporation and thermal behavior. Under the conditions investigated, CNF exhibited higher degrees of substitution than linter cellulose and microcrystalline cellulose, while the consolidation route was shown to affect reactivity through hornification effects. Taken together, these findings link precursor structure, dehydration strategy, and nitration outcome within a single experimental framework and delimit the conditions under which nanocellulose-based nitrocellulose can be meaningfully evaluated as a morphology-controlled energetic material.
